# Comprehensive analysis of an endoplasmic reticulum stress-related gene prediction model and immune infiltration in idiopathic pulmonary fibrosis

**DOI:** 10.3389/fimmu.2023.1305025

**Published:** 2024-01-11

**Authors:** Honglan Zhu, Aiming Zhou, Menglin Zhang, Lin Pan, Xiao Wu, Chenkun Fu, Ling Gong, Wenting Yang, Daishun Liu, Yiju Cheng

**Affiliations:** ^1^ Department of Respiratory and Critical Care Medicine, The Affiliated Hospital of Guizhou Medical University, Guiyang, China; ^2^ Department of Clinical Medicine, Guizhou Medical University, Guiyang, China; ^3^ Department of Respiratory and Critical Care Medicine, The Third Affiliated Hospital (The First People’s Hospital of Zunyi) of Zunyi Medical University, Zunyi, China; ^4^ Department of Cardiac Surgery, The Affiliated Hospital of Guizhou Medical University, Guiyang, China; ^5^ Department of Respiratory and Critical Care Medicine, The First People’s Hospital of Anshun, Anshun, China; ^6^ Department of Respiratory and Critical Care Medicine, The Second Affiliated Hospital of Army Medical University, Chongqing, China; ^7^ Department of Respiratory and Critical Care Medicine, The Second People’s Hospital of Guiyang, Guiyang, China; ^8^ Department of Clinical Medicine, Zunyi Medical University, Zunyi, China; ^9^ Department of Respiratory and Critical Care Medicine, The Fourth People’s Hospital of Guiyang, Guiyang, China

**Keywords:** idiopathic pulmonary fibrosis, endoplasmic reticulum stress (ER stress), diagnosis, prognosis, bioinformatics analysis, immune filtration

## Abstract

**Background:**

Idiopathic pulmonary fibrosis (IPF) is a chronic progressive interstitial lung disease. This study aimed to investigate the involvement of endoplasmic reticulum stress (ERS) in IPF and explore its correlation with immune infiltration.

**Methods:**

ERS-related differentially expressed genes (ERSRDEGs) were identified by intersecting differentially expressed genes (DEGs) from three Gene Expression Omnibus datasets with ERS-related gene sets. Gene Set Variation Analysis and Gene Ontology were used to explore the potential biological mechanisms underlying ERS. A nomogram was developed using the risk signature derived from the ERSRDEGs to perform risk assessment. The diagnostic value of the risk signature was evaluated using receiver operating characteristics, calibration, and decision curve analyses. The ERS score of patients with IPF was measured using a single-sample Gene Set Enrichment Analysis (ssGSEA) algorithm. Subsequently, a prognostic model based on the ERS scores was established. The proportion of immune cell infiltration was assessed using the ssGSEA and CIBERSORT algorithms. Finally, the expression of ERSRDEGs was validated *in vivo* and *in vitro* via RT-qPCR.

**Results:**

This study developed an 8-ERSRDEGs signature. Based on the expression of these genes, we constructed a diagnostic nomogram model in which agouti-related neuropeptide had a significantly greater impact on the model. The area under the curve values for the predictive value of the ERSRDEGs signature were 0.975 and 1.000 for GSE70866 and GSE110147, respectively. We developed a prognostic model based on the ERS scores of patients with IPF. Furthermore, we classified patients with IPF into two subtypes based on their signatures. The RT-qPCR validation results supported the reliability of most of our conclusions.

**Conclusion:**

We developed and verified a risk model using eight ERSRDEGs. These eight genes can potentially affect the progression of IPF by regulating ERS and immune responses.

## 1 Introduction

Idiopathic pulmonary fibrosis (IPF) is a chronic and progressive interstitial lung disease that has a high mortality rate and limited treatment options (1). The average survival time after diagnosis is only 2–3 years (2). Although two Food and Drug Administration-approved drugs, pirfenidone and nintedanib, can slow the decline in lung function, they do not halt disease progression or reduce mortality (3). Currently, IPF is diagnosed by identifying a specific pattern of lung damage known as usual interstitial pneumonia using high-resolution computed tomography or lung biopsy (4). Despite advancements in imaging technology and disease classification systems, diagnosis remains challenging. Certain serum biomarkers, such as surfactant protein A/D, matrix metallopeptidase-7, periostin, osteopontin, and chemokine ligand 18, have shown predictive value for diagnosis, prognosis, and treatment response (5, 6). However, the heterogeneity of IPF and the involvement of various pathophysiological factors in its progression make its diagnosis and treatment difficult. Therefore, there is a pressing need for more accurate, noninvasive, and feasible markers that could aid in the diagnosis and prognosis of IPF, allowing for more precise and personalized treatment approaches for patients with this condition.

The endoplasmic reticulum (ER) is an essential organelle that maintains proteostasis, or the balance between protein production and degradation within cells (7). ER stress (ERS) is a cellular response that occurs when misfolded proteins accumulate in the ER and disrupt protein homeostasis (8). Several factors, such as the expression of mutant proteins, oxidative stress, impaired autophagy, mechanical stretch, hypoxia, and aging, can induce ERS in IPF (9). Emerging evidence suggests that ERS plays a critical role in IPF by regulating the apoptosis and senescence of alveolar epithelial cells (AECs) (10), promoting epithelial-mesenchymal transition (11) and facilitating myofibroblast differentiation (12). Specifically, a fibroblast-enriched ER protein called TXNDC5 promotes pulmonary fibrosis by enhancing transforming growth factor-β (TGF-β) signaling through stabilizing TGFBR1 (13). However, the exact relationship between ERS and IPF has not been fully elucidated.

Recently, there has been growing interest in the role of immune infiltration in cancer research. However, it is important to note that immune dysregulation also plays a crucial role in the development of IPF. Various immune cells are involved in the pathogenesis of pulmonary fibrosis. When AECs are continuously injured, alveolar macrophages release profibrotic cytokines and chemokines, such as CCL18, CHI3L1, MMPs, Wnt, and TGF-β (14). These substances activate fibroblasts, which differentiate into myofibroblasts. Myofibroblasts produce an extracellular matrix (ECM), leading to the thickening of the pulmonary interstitium. Regulatory T cells inhibit Th1 cell activation, which alters the Th1/Th2 balance in the lungs. Th2 cells produce IL-4 and IL-13, which promote the polarization of alveolar macrophages into a profibrotic M2 phenotype and differentiation of circulating fibrocytes into fibroblasts (15). Studies have shown that ERS regulates the polarization of M2 macrophages in the lungs (16, 17). However, the precise mechanism underlying this relationship is poorly understood. Furthermore, whether ERS is involved in interactions between other immune cells in IPF is unclear.

This study successfully established a connection between ERS and immune infiltration in the context of IPF through a comprehensive set of methodical bioinformatics analyses. Furthermore, a diagnostic and prognostic prediction model and a subclass classification system for patients with IPF were developed based on genes associated with ERS. This study contributes substantially to advancing knowledge regarding the intricate relationships among ERS, immune infiltration, and IPF pathogenesis.

## 2 Materials and methods

### 2.1 Acquisition of IPF datasets and ERS-related genes

The raw gene expression profiles of patients with IPF were acquired from various datasets on the Gene Expression Omnibus (GEO) website (https://www.ncbi.nlm.nih.gov/geo/) (18). The following datasets were used: GSE70866 (19), GSE28042 (20), GSE110147 (21), GSE24206 (22), and GSE93606 (23). The R package “GEOquery”(version 2.68.0) (24) was utilized to access these datasets. The GSE70866 dataset contains 20 healthy individuals and 212 IPF bronchoalveolar lavage fluid (BALF) specimens. These specimens were analyzed using the Agilent-028004 SurePrint G3 Human GE 8×60K Microarray on the GPL14550 platform.

The GSE28042 dataset consists of 19 healthy subjects and 75 IPF peripheral blood mononuclear cell (PBMC) specimens. These specimens were analyzed using the Agilent-014850 Whole Human Genome Microarray 4×44K G4112F on the GPL6480 platform.

The GSE110147 dataset included 11 healthy, 15 nonspecific interstitial pneumonia, and 22 IPF fresh-frozen lung tissue samples. The samples were analyzed using the Affymetrix Human Gene 1.0 ST Array on the GPL6244 platform. Fifteen nonspecific interstitial pneumonia samples were excluded from further analysis. The GSE70866, GSE28042, and GSE110147 datasets were used as training sets. The details of these datasets are listed in **Table 1**.

**Table 1:** Idiopathic Pulmonary Fibrosis data set information list.

	GSE70866	GSE28042	GSE110147
Platform	GPL14550	GPL6480	GPL6244
Species	Homo sapiens	Homo sapiens	Homo sapiens
Tissue	bronchoalveolar lavage cell	peripheral blood mononuclear cell	Fresh frozen lung tissue specimens
Samples in IPF group	112	75	22
Samples in Control group	20	19	11
Reference	BAL Cell Gene Expression Is Indicative of Outcome and Airway Basal Cell Involvement in Idiopathic Pulmonary Fibrosis	Peripheral blood mononuclear cell gene expression profiles predict poor outcome in idiopathic pulmonary fibrosis	Comprehensive gene expression profiling identifies distinct and overlapping transcriptional profiles in non-specific interstitial pneumonia and idiopathic pulmonary fibrosis

IPF, Idiopathic Pulmonary Fibrosis; GEO, Gene Expression Omnibus.

The GSE24206 dataset comprised six healthy donors and 17 IPF lung tissue specimens. These specimens were analyzed using the Affymetrix Human Genome U133 Plus 2.0 Array on the GPL570 platform. The GSE93606 dataset included 20 healthy controls and 154 IPF BALF and PBMC samples. The samples were analyzed using the Affymetrix Human Gene 1.1 ST Array on the GPL11532 platform. The GSE24206 and GSE93606 datasets were selected as external validation sets. Details of these datasets are provided in **Supplementary Table S1**.

A total of 2,269 ERSRGs were obtained from the GeneCards database (25) (https://www.genecards.org/). Additionally, 33 ERSRGs were identified from published literature available on the PubMed website (https://pubmed.ncbi.nlm.nih.gov) (26, 27). After removing duplicate genes, 2,279 unique ERSRGs were included in subsequent analyses, as shown in **Supplementary Table S2**.

### 2.2 Screening of ERS-related differentially expressed genes

The raw microarray data from three datasets, GSE70866, GSE28042, and GSE110147, were subjected to preprocessing for quality control using the “limma” R package (version 3.56.2) (28). This involved background adjustments and normalization. The resulting expression matrices before and after normalization were visualized using boxplots. The “limma” R package (version 3.56.2) was also employed to identify DEGs between the IPF patient and control groups. The threshold values for significance were set at an adjusted p value (P.adj)< 0.05 and |log2 fold change| > 0.5 in all three datasets. The results were visualized using the “ggplot2” and “pheatmap” R packages (version 1.0.12). Common DEGs (co-DEGs) were identified by intersecting the DEGs from the three datasets. Subsequently, the co-DEGs were further intersected with ERSRGs to determine the ERSRDEGs. Co-DEGs and ERSRDEGs were identified using an online program, and Venn diagrams were generated to illustrate overlapping genes.

### 2.3 Functional annotation of ERSRDEGs and IPF datasets

Gene Ontology (GO) analysis was conducted to assess the biological functions of ERSRDEGs. The analyses included the evaluation of biological processes (BP), molecular functions (MF), and cellular components (CC). The R package “ClusterProfiler” (version 4.8.3) (29) was utilized for this analysis. A significance threshold of p.adj< 0.05 and false discovery rate (FDR)< 0.05 were applied to determine significant enrichment.

To further evaluate the relationship between genes in a predefined gene set and a specific phenotype, gene set enrichment analysis (GSEA) (30) was employed. This analysis was performed on datasets related to IPF. The gene set “c2.cp.all.v2022.1. Hs.symbols.gmt” from the MSigDB database (25) was used for GSEA, and only terms with p.adj< 0.05 and FDR< 0.05 were considered significant.

### 2.4 Weighted gene coexpression network analysis

A weighted gene coexpression network analysis (WGCNA) (31) was conducted on the DEGs between individuals with IPF and control individuals in the GSE70866 dataset. The analysis employed the WGCNA R package (32) with the RsquaredCut parameter set to 0.90, the minimum number of module genes set to 100, and the module merge cut height set to 0.2. This package constructs scale-free coexpression networks for clinical phenotypes. Hierarchical clustering analysis was employed to filter the discrete cases. Subsequently, an appropriate soft power was selected to construct a weighted adjacency matrix, which was then transformed into a topological overlap matrix (TOM). The TOM contained module assignments labeled with color and module features (ME). Pearson’s correlation coefficients were calculated to evaluate the relationship between ME and the clinical features. Finally, the genes in the most significant module associated with IPF were intersected with ERSRDEGs for further investigation.

### 2.5 Co-ERSRDEG-associated diagnostic model construction

In this study, we initially developed a Support Vector Machine (SVM) model using the SVM algorithm (33) to identify ERSRDEGs associated with IPF. The number of ERSRDEGs was selected based on accuracy and error rate. Additionally, we employed the random forest (34) (RF) algorithm, which utilizes bootstrap aggregation and randomization of predictors to further screen candidate ERSRDEGs for IPF diagnosis. The RF model was implemented using the “randomForest” package (version 4.7-1.1) (35) in R, with the following parameters: ntrees = “1000” and “set.seed (234)”. The number of trees and error value of the 10-fold cross-validation were plotted on the X and Y axes. Subsequently, a binary logistic regression analysis was conducted to investigate the impact of the selected ERSRDEGs on IPF. The logistic regression model was visualized using the Forest Plot. To prevent overfitting, we employed the least absolute shrinkage and selection operator (36) (LASSO) regression algorithm to identify the ERSRDEGs with the highest predictive value for IPF in the logistic regression model. The “glmnet” (37) package (version 4.1-8) in R was utilized for this analysis. The ERSRDEGs and their coefficients were determined using the best penalty parameter λ, which was associated with the smallest 10-fold cross-validation error. The results of the LASSO regression analysis were presented using a diagnostic model and variable locus diagrams. Finally, the identified genes were used to construct an optimal risk signature, which was determined by a linear combination of their expression levels, weighted with the regression coefficients from the LASSO analysis. The risk score for each sample was calculated as follows:


$$riskScore\;=\;\sum_{i}Coefficient\;\left(hub\;gene_{i}\right)*mRNA\;Expression\;\left(hub\;gene_{i}\right)$$

Finally, the ERSRDEGs identified by the SVM, RF, and Logistic-LASSO models were compared using a Venn diagram to identify overlapping ERSRDEGs. A nomogram (38) was constructed using the “rms” package (version 6.7-1) in R based on the co-ERSRDEGs to visualize the diagnostic model for IPF. The model accuracy was assessed by evaluating its predictive value using a calibration curve. Furthermore, the clinical impact of the model’s judgment on patients with IPF was quantified using decision curve analysis (DCA) (39) by plotting a clinical impact curve.

### 2.6 Validation of expression of co-ERSRDEGs and effect of the diagnostic prediction model

The expression matrix of co-ERSRDEGs was obtained from four datasets: GSE70866, GSE28042, GSE110147, and GSE24206. The expression differences between IPF and healthy samples were assessed using the Wilcoxon rank sum test and visualized using the “ggplot2” package. Statistical significance was set at p< 0.05. To evaluate the accuracy of the model’s predictions, receiver operating characteristic (ROC) (40) curves and corresponding area under the curve (AUC) values were calculated using the “survivalROC” R package. The diagnostic model based on co-ERSRDEGs was tested in the training sets GSE70866 and GSE110147, as well as the validation sets GSE24206 and GSE93606.

### 2.7 Spearman correlation analysis of co-ERSRDEGs

In this study, Spearman correlation analysis was conducted to examine the expression levels of co-ERSRDEGs in the GSE70866 dataset. The analysis was performed using the “limma” package (28), with a significance threshold of |R| > 0.2 and p< 0.05. Scatter plots were generated to visualize the results using the “ggplot2,” “ggpubr,” and “ggExtra” packages.

### 2.8 Construction of ERS score prognostic model

Initially, single-sample GSEA (ssGSEA) was employed to quantify the ERS phenotype scores of all samples in the GSE70866 dataset. This was performed by utilizing the expression matrix of co-ERSRDEGs and the “GSVA” package (41). Subsequently, the patients with IPF were divided into high- and low-risk groups based on the median ERS score obtained from the GSE70866 dataset. The overall survival (OS) of these two groups was compared via Kaplan–Meier analysis and the log-rank test. To assess the prognostic value of the risk model, ROC analysis, time-dependent ROC curve analysis, and calculation of the AUC values were conducted. These evaluations were performed to determine the accuracy of the model for predicting patient outcomes.

### 2.9 Classification of IPF subtypes

The “ConsensusClusterPlus” (35) package in R was employed to cluster the patients with IPF in the GSE70866 dataset. This clustering was based on the expression of co-ERSRDEGs between patients with IPF and controls to identify the molecular subtypes of IPF. The analysis was conducted using the following parameters: maxK = 8, reps = 50, pItem = 0.8, pFeature = 1, clusterAlg = “pam,” and distance = “spearman.” The outputs included consensus cumulative distribution function (CDF) plots and the relative change in the area under the CDF curve. Principal component analysis (PCA) was conducted to further validate gene expression patterns in the identified clusters. Additionally, Kaplan–Meier survival analysis using the survival package was employed to assess the differences in OS between the various subtypes of IPF.

### 2.10 Immune infiltration and correlation between co-ERSRDEGs and immune cells

This study divided IPF cases in the GSE70866 dataset into two groups based on their risk scores obtained from the LASSO regression analysis. Similarly, patients were divided into high and low ERS score groups based on their ERS phenotype scores. The relative abundances of 28 immune cell types in patients with IPF were quantified using the ssGSEA algorithm (42, 43). The differences in immune cell abundance between the risk score groups, ERS score groups, and IPF subtypes were analyzed using the Mann–Whitney U test and presented using boxplots. The correlation between the abundance of immune cell infiltration in different groups and subtypes was assessed using Pearson’s correlation analysis and visualized using a correlation matrix plot created with the “ggplot2” R package. Pearson’s correlation analysis was used to examine the relationship between the abundance of immune cell infiltration and expression of co-ERSRDEGs. The results were displayed using a correlation dot plot generated with the “ggplot2” R package. Additionally, the CIBERSORT algorithm (44) was used to calculate the infiltration fraction of 22 immune cell types in the risk score groups, ERS score groups, and IPF subtypes. The results were presented as histograms. Differences in immune cell abundance between different groups were analyzed using the Wilcoxon rank-sum test and are displayed using boxplots. The correlation between the abundance of immune cell infiltration and co-ERSRDEG expression was assessed using Pearson’s correlation analysis and visualized using a correlation matrix plot created with the “ggplot2” R package.

### 2.11 Preliminary validation of expression of co-ERSRDEGs in ERS A549 cell model and embryonic mouse fibroblasts 3T3 cell model

A549 and embryonic mouse fibroblast 3T3 cell lines were obtained from the American Type Culture Collection (ATCC). The cells were maintained in specific media; DMEM and Ham’s F-12K were used for embryonic mouse fibroblast 3T3 and A549 cells respectively. The media were supplemented with 10% fetal bovine serum and 1% penicillin-streptomycin. The cells were cultured at 37°C with 5% v/v CO2. The study consisted of control and treatment groups. The treatment group was exposed to 1 μg/mL tunicamycin (MCE, NJ, USA) for 24 h in embryonic mouse fibroblast 3T3 cells and 4 μg/mL tunicamycin for 48 hours in A549 cells. RNA was then extracted from the cells for RT-qPCR. The relative gene expression was determined using the **Equation 2** ^-ΔΔct. Primer sequences are listed in **Supplementary Table S3**.

### 2.12 Validation of expression of co-ERSRDEGs in bleomycin-induced pulmonary fibrosis mouse model

Male C57BL/6 mice aged 6-9 weeks, with an average weight of 20-25 g, were obtained from the Laboratory Animal Center of Guizhou Medical University. The study protocol was reviewed and approved by the Animal Ethics Committee of the institution. To induce fibrotic changes, the experimental mice received 50 μL of bleomycin (5 mg/kg) via intratracheal administration, whereas the control mice were administered an equal volume of phosphate buffer (PBS). After 21 days, lung tissue samples were collected. A portion of the sample was used for subsequent RT-qPCR. In contrast, the remaining portion was subjected to hematoxylin-eosin (HE) and Masson staining following the instructions in the staining kit.

### 2.13 Statistical analysis

Statistical analysis was performed using R software (version 4.2.2) and the corresponding packages. All data were expressed as the mean ± standard deviation (SD). Gene expression in the two groups was compared using Student’s t-test, Wilcoxon test, or Mann–Whitney U test, where appropriate. Correlation analyses were performed using Spearman’s or Pearson’s correlation analyses. GraphPad software (version 8.0) was used to visualize statistical results. Two-tailed p<0.05 was considered significant.

**Figure 1:**
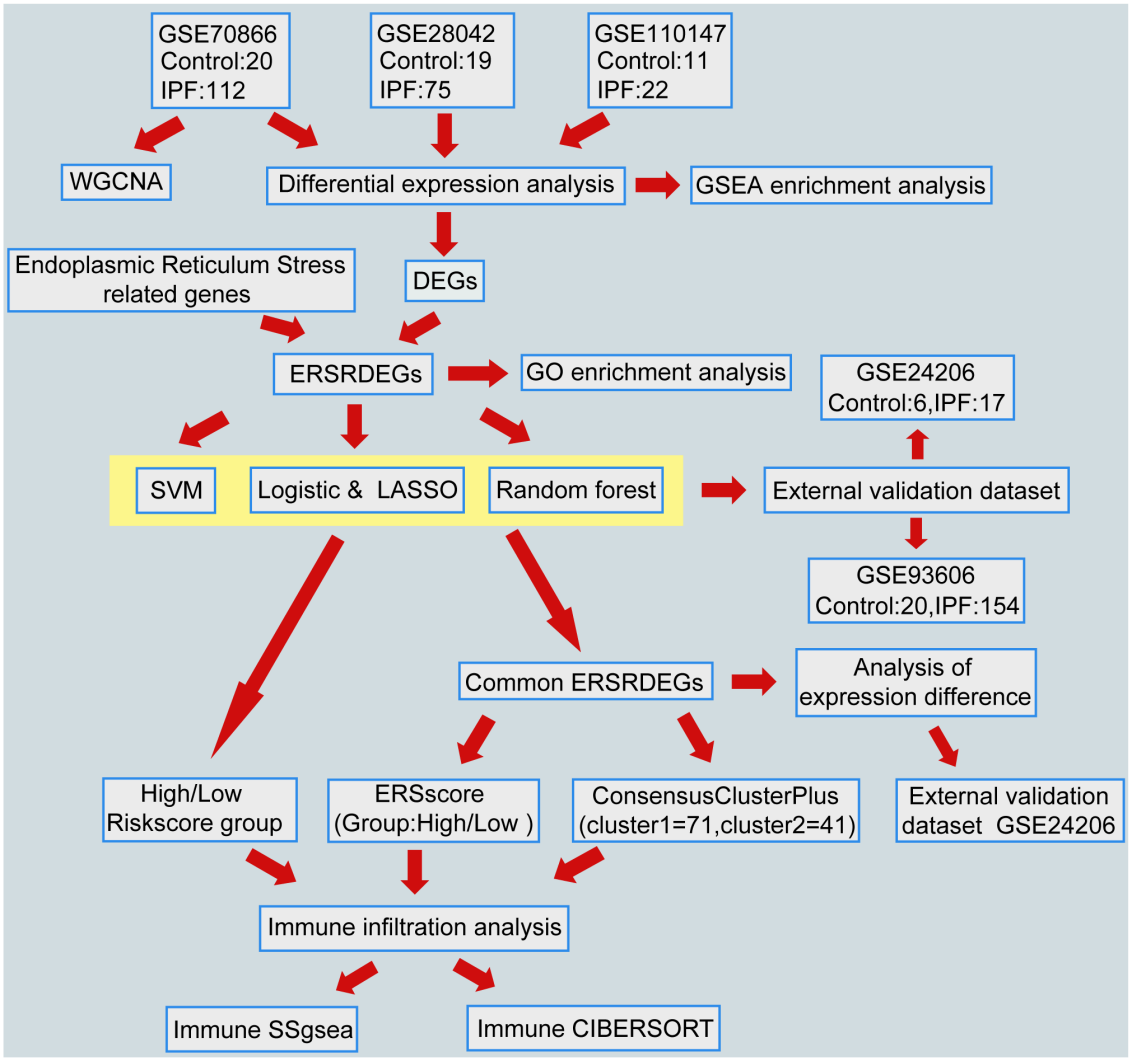
Flowchart of data analysis. IPF, Idiopathic Pulmonary Fibrosis; WGCNA, Weighted gene coexpression network analysis; DEGs, differentially expressed genes; ERSRDEGs, endoplasmic reticulum stress-related DEGs; SVM, support vector machine; LASSO, least absolute shrinkage and selection operator; GSEA, gene set enrichment analysis; GO, gene ontology; ERS score, endoplasmic reticulum stress scores; ssGSEA, single-sample gene set enrichment analysis.

A flowchart representing the overall concepts and procedures employed in this study is shown in **Figure 1**.

## 3 Results

### 3.1 Identification of DEGs and ERSRDEGs between IPF and control

**Figure 2:**
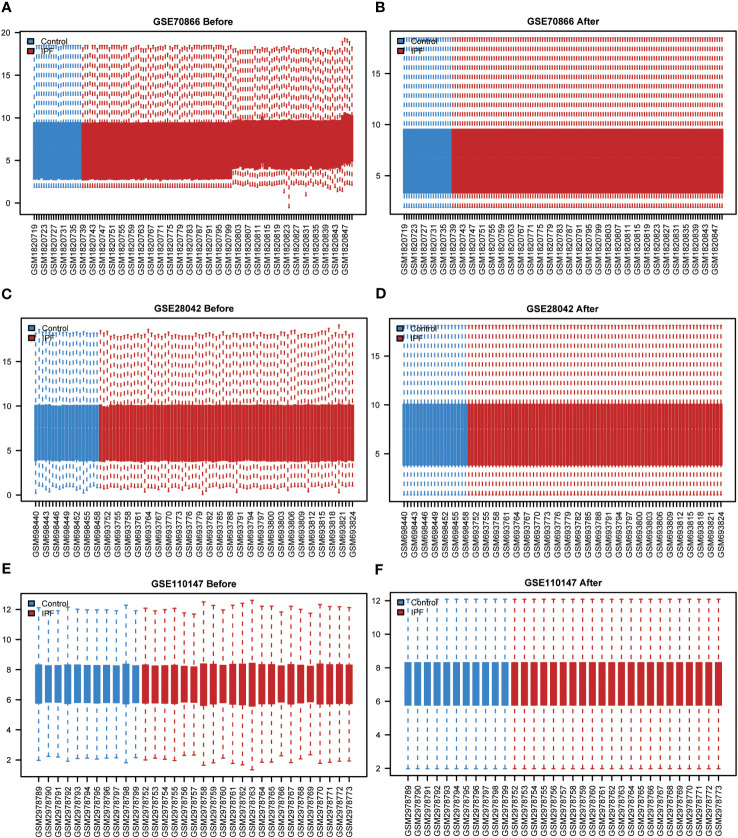
Standardized processing of IPF datasets. **(A)** Boxplot of the GSE70866 data prior to normalization. **(B)** Boxplot of the GSE70866 data post normalization. **(C)** Boxplot of the GSE28042 data prior to normalization. **(D)** Boxplot of the GSE28042 data post normalization. **(E)** Boxplot of the GSE110147 data prior to normalization. **(F)** Boxplot of the GSE110147 data post normalization. IPF, idiopathic pulmonary fibrosis.

**Figure 3:**
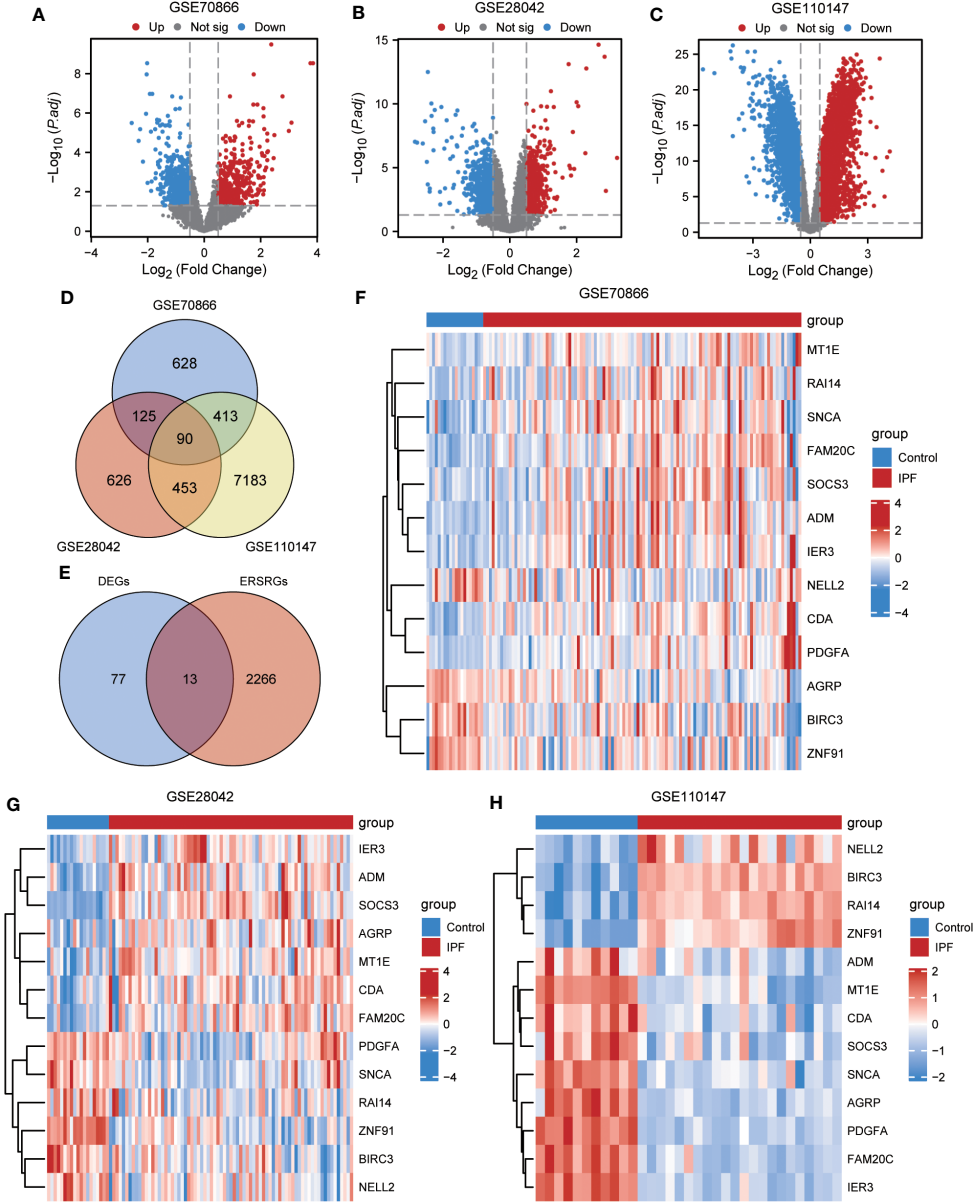
Differential expression analysis of IPF datasets. **(A, B)** Volcano plot of DEGs in the cohort of GSE70866 **(A)**, GSE28042 **(B)** and GSE110147 **(C)**. **(D)** Venn diagram illustrating overlapping genes among DEGs identified in GSE110147, GSE28042, and GSE110147. **(E)** €Intersection of 90 co-DEGs with 2279 ERS-related genes. F-H Clustering heatmap of ERSRDEGs in GSE70866 dataset **(F)**, GSE28042 dataset **(G)**, and GSE110147 dataset **(H)**. IPF, idiopathic pulmonary fibrosis; DEGs, differentially expressed genes; ERS, endoplasmic reticulum stress; ERSRDEGs, ERS-related DEGs.

To ensure the comparability of gene expression data across samples, the gene expression levels of the GSE70866, GSE28042, and GSE110147 datasets were normalized, and batch effects were subsequently eliminated (**Figures 2A–F**). Furthermore, the “limma” package was used to examine DEGs in patients with IPF and healthy controls using p-value<0.05 and |log2FC|>0.5 as thresholds. A total of 1,256 DEGs were identified in the GSE70866 cohort of the GEO database. Among these genes, 499 were upregulated, and 757 were downregulated. Similarly, the GSE28042 dataset revealed 1,294 DEGs, of which 608 were upregulated and 686 were downregulated. Additionally, the GSE110147 dataset contained 8,139 DEGs, with 4,211 upregulated and 3,928 downregulated genes. **Figures 3A–C** depict the expression patterns of DEGs using heatmap visualization. DEGs from three datasets, GSE70866, GSE28042, and GSE110147, were compared, and co-DEGs were identified. A total of 90 co-DEGs were shared among these datasets, as illustrated in a Venn diagram (**Figure 3D**). A collection of 2,269 ERSRGs was acquired from the GeneCards database and PubMed. These genes intersected with the co-DEGs, resulting in 13 overlapping ERSRDEGs, which were further examined. **Figure 3E** shows the 13 ERSRDEGs (ADM, AGRP, BIRC3, CDA, FAM20C, IER3, MT1E, NELL2, PDGFA, RAI14, SNCA, SOCS3, and ZNF91) in a Venn diagram. The expression patterns of these genes in the GSE70866, GSE28042, and GSE110147 datasets are represented using heat maps, as shown in **Figures 3, F–H**, respectively.

### 3.2 Functional annotation of ERSRDEGs

**Figure 4:**
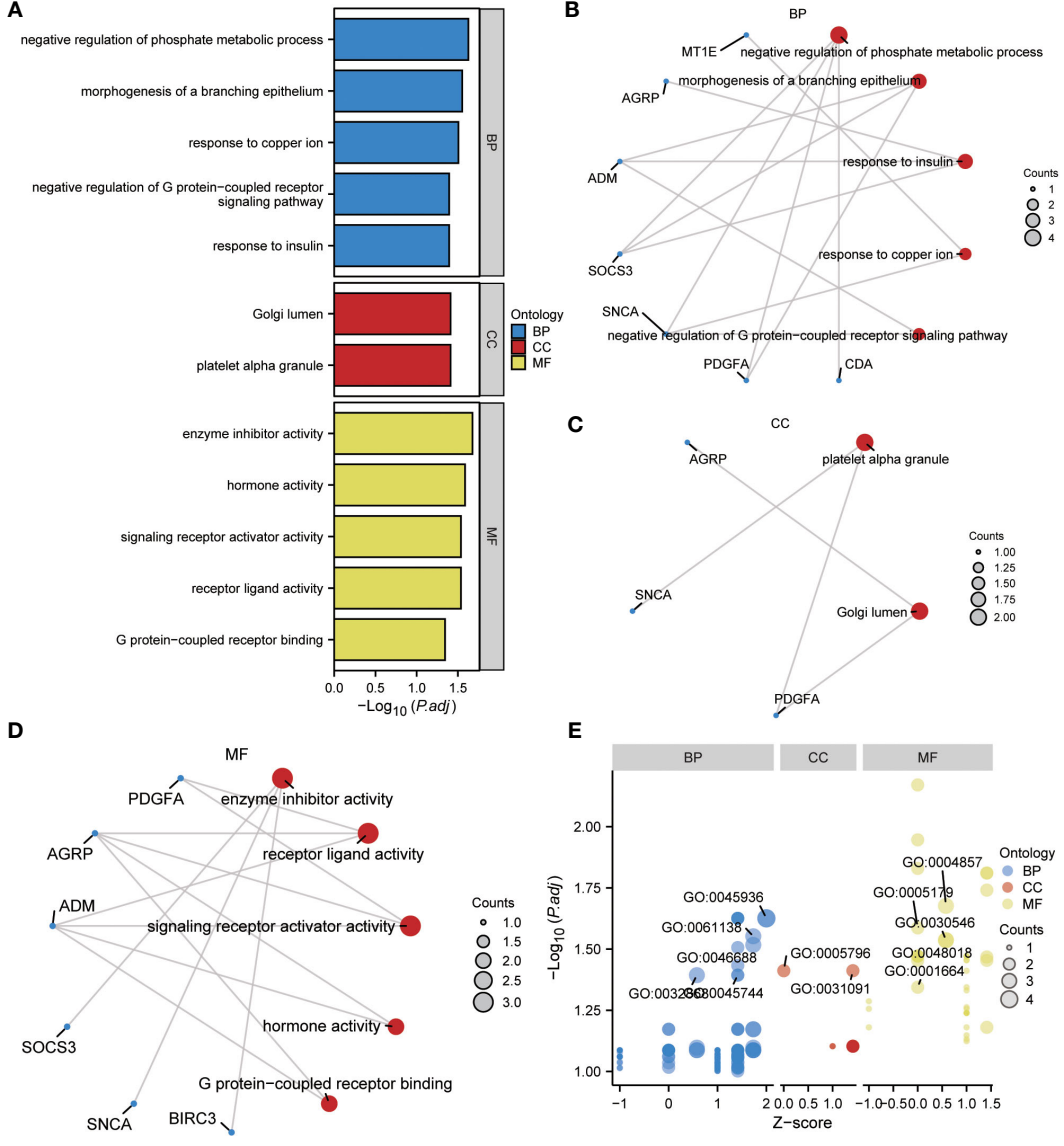
Results of GO enrichment analysis using ERSRDEGs. **(A)** Bar chart showing GO enrichment analysis of ERSRDEGs, including BP, CC, and MF. **(B-D)** Ring network diagram showing GO enrichment analysis of ERSRDEGs, including BP **(B)**, CC **(C)**, and MF **(D)**. Red circles and blue dots indicate pathways and specific genes, respectively. **(E)** Bubble chart showing combined enrichment analysis of log FC and GO of ERSRDEGs. The blue, red, and yellow circles represent the BP, CC, and MF, respectively. GO, gene ontology; BP, biological process; CC, cellular component; MF, molecular function; ERSRDEGs, endoplasmic reticulum stress-related differentially expressed genes.

To gain further insight into the biological function of the 13 ERSRDEGs (ADM, AGRP, BIRC3, CDA, FAM20C, IER3, MT1E, NELL2, PDGFA, RAI14, SNCA, SOCS3, and ZNF91) in IPF, we performed GO enrichment analyses (**Table 2**). GO analysis encompassed three categories: BP, CC, and MF. In terms of BP, ERSRDEGs were predominantly enriched in negative regulation of phosphate metabolism, morphogenesis of a branching epithelium, response to insulin, response to copper ion, and negative regulation of the G protein-coupled receptor signaling pathway (**Figures 4A, B**). With regard to CC, the top two enriched were platelet alpha granule and Golgi lumen (**Figures 4A, C**). In the context of MF, ERSRDEGs were closely associated with enzyme inhibitor activity, receptor-ligand activity, signaling receptor activator activity, hormone activity, and G protein-coupled receptor binding (**Figures 4A, D**). Furthermore, a combined GO and log FC enrichment analysis revealed that ERSRDEGs were primarily enriched in BP pathways, as visualized in a bubble diagram (**Figure 4E**).

**Table 2:** GO enrichment Analysis results of ERSRDEGs.

ONTOLOGY	ID	Description	GeneRatio	BgRatio	pvalue	p.adjust	qvalue
BP	GO:0045936	negative regulation of phosphate metabolic process	4/13	440/18800	0.000179	0.023676	0.013237
BP	GO:0061138	morphogenesis of a branching epithelium	3/13	185/18800	0.000249	0.028034	0.015674
BP	GO:0032868	response to insulin	3/13	259/18800	0.000667	0.040401	0.022587
BP	GO:0046688	response to copper ion	2/13	41/18800	0.000356	0.031172	0.017428
BP	GO:0045744	negative regulation of G protein-coupled receptor signaling pathway	2/13	54/18800	0.000619	0.040401	0.022587
CC	GO:0031091	platelet alpha granule	2/13	91/19594	0.001609	0.038756	0.026462
CC	GO:0005796	Golgi lumen	2/13	104/19594	0.002095	0.038756	0.026462
MF	GO:0004857	enzyme inhibitor activity	3/13	390/18410	0.002304	0.021062	0.008314
MF	GO:0048018	receptor ligand activity	3/13	489/18410	0.004367	0.029086	0.011481
MF	GO:0030546	signaling receptor activator activity	3/13	496/18410	0.004545	0.029086	0.011481
MF	GO:0005179	hormone activity	2/13	122/18410	0.003239	0.025911	0.010228
MF	GO:0001664	G protein-coupled receptor binding	2/13	288/18410	0.016977	0.045272	0.017871

GO, Gene Ontology; BP, biological process; CC, cellular component; MF, molecular function; ERSRDEGs, Endoplasmic reticulum stress related differentially expressed genes.

### 3.3 GSEA of IPF datasets

**Figure 5:**
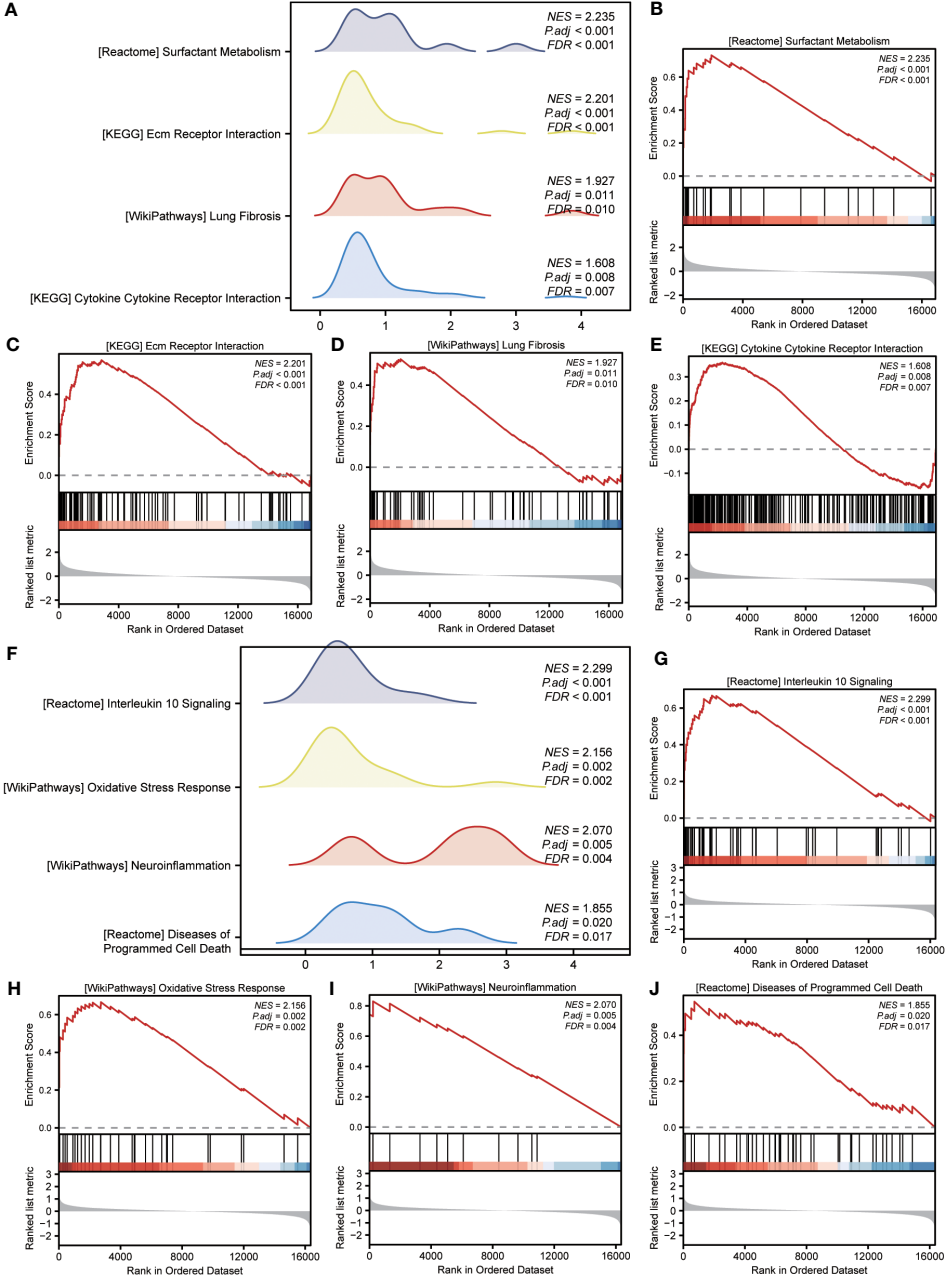
Enrichment plots from GSEA in GSE70866 and GSE28042 datasets. **(A, F)** Mountain maps showing the GSEA results of GSE70866 **(A)** and GSE28042 **(F)** datasets. **(B–E)** Enrichment showed that DEG function mainly focused on surfactant metabolism **(B)**, ECM receptor interaction **(C)**, lung fibrosis **(D)**, and cytokine-cytokine receptor interaction **(E)** in the GSE70866 dataset. **G-J** Enrichment showed that DEGs function mainly focused on interlerkin-10 signaling **(G)**, oxidative stress response **(H)**, neuroinflammation **(I)**, and diseases of programmed cell death**(J)** in the GSE28042 dataset. GSEA, gene set enrichment analysis; DEGs, differentially expressed genes; ECM, extracellular matrix.

**Figure 6:**
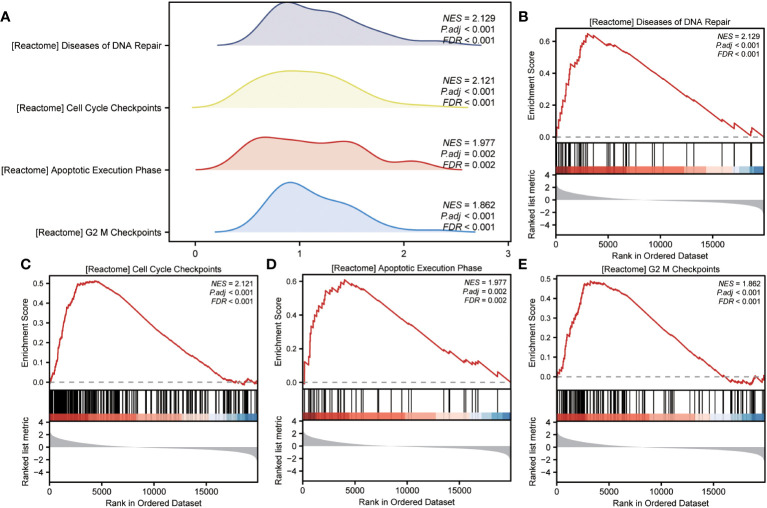
Enrichment plots from GSEA in GSE110147 dataset. **(A)** Mountain maps showing the GSEA results of the GSE110147 dataset. **(B–E)** Enrichment showed that DEGs function mainly focused on the diseases of DNA repair **(B)**, cell cycle checkpoints **(C)**, apoptotic execution phase **(D)**, and G2 M checkpoints **(E)**. GSEA, gene set enrichment analysis; DEGs, differentially expressed genes.

GSEA was performed to examine the relationship between gene expression and the BP, CC, and MF implicated in patients with IPF compared with healthy controls. The GSE70866, GSE28042, and GSE110147 datasets were used for this analysis. To identify significant enrichment, the enrichment screening criteria were set at a p.adj< 0.05 and FDR value (q.Vue)< 0.05. Results showed a significant concentration of genes associated with various pathways in patients with IPF compared with the control group. Specifically, in the GSE70866 dataset, the genes were concentrated in pathways such as surfactant metabolism, ECM receptor interaction, lung fibrosis, and cytokine-cytokine receptor interaction. These results are shown in **Figures 5B–E** and **Table 3**, as well as in the mountain maps in **Figure 5A**. Similarly, in the GSE28042 dataset, genes were significantly enriched in pathways, including interlerkin-10 signaling, oxidative stress response, neuroinflammation, and diseases of programmed cell death. These findings are shown in **Figures 5G–J** and **Table 4**, along with mountain maps in **Figure 5F**. Furthermore, in the GSE110147 dataset, genes were notably enriched in pathways, such as the diseases of DNA repair, cell cycle checkpoints, apoptotic execution phase, and G2 M checkpoints. These results are presented in **Figures 6B–E** and **Table 5**, as well as in the mountain maps in **Figure 6A**.

**Table 3:** GSEA enrichment analysis results of IPF dataset GSE70866.

Description	setSize	enrichmentScore	NES	pvalue	p.adjust	qvalue
REACTOME_SURFACTANT_METABOLISM	25	0.73283	2.23456	0.00001	0.00063	0.00055
KEGG_ECM_RECEPTOR_INTERACTION	84	0.56826	2.20147	0.00000	0.00002	0.00002
WP_OSTEOPONTIN_SIGNALING	13	0.84762	2.18875	0.00002	0.00129	0.00112
REACTOME_RESPONSE_TO_METAL_IONS	14	0.82312	2.15248	0.00002	0.00165	0.00144
NABA_CORE_MATRISOME	258	0.47570	2.13729	0.00000	0.00000	0.00000
WP_LUNG_FIBROSIS	60	0.52739	1.92654	0.00027	0.01103	0.00961
KEGG_CYTOKINE_CYTOKINE_RECEPTOR_INTERACTION	248	0.35950	1.60817	0.00019	0.00844	0.00735
WP_VITAMIN_D_RECEPTOR_PATHWAY	177	0.37463	1.60547	0.00077	0.02276	0.01983
KEGG_FOCAL_ADHESION	196	0.35995	1.57129	0.00123	0.03275	0.02853
REACTOME_DISEASES_OF_METABOLISM	237	0.34926	1.55973	0.00099	0.02830	0.02466
WP_BARDETBIEDL_SYNDROME	75	-0.50629	-2.02919	0.00001	0.00089	0.00078
REACTOME_TCR_SIGNALING	116	-0.48146	-2.06927	0.00000	0.00005	0.00004
REACTOME_PD_1_SIGNALING	23	-0.68568	-2.07357	0.00005	0.00340	0.00296
WP_CILIOPATHIES	144	-0.48414	-2.15279	0.00000	0.00000	0.00000
WP_JOUBERT_SYNDROME	70	-0.55116	-2.16270	0.00000	0.00015	0.00013

GSEA, Gene Set Enrichment Analysis; IPF, Idiopathic Pulmonary Fibrosis.

**Table 4:** GSEA enrichment analysis results of IPF dataset GSE28042.

Description	setSize	enrichmentScore	NES	pvalue	p.adjust	qvalue
REACTOME_INTERLEUKIN_10_SIGNALING	44	0.66838	2.29882	0.00000	0.00009	0.00008
WP_OXIDATIVE_STRESS_RESPONSE	33	0.66638	2.15634	0.00002	0.00199	0.00168
PID_ATF2_PATHWAY	56	0.58284	2.12268	0.00001	0.00116	0.00098
PID_FOXM1_PATHWAY	39	0.63297	2.10475	0.00003	0.00262	0.00222
WP_ZINC_HOMEOSTASIS	37	0.62934	2.07420	0.00005	0.00365	0.00309
BIOCARTA_ETS_PATHWAY	17	0.74879	2.07264	0.00009	0.00554	0.00470
WP_NEUROINFLAMMATION	12	0.83011	2.07013	0.00007	0.00483	0.00409
REACTOME_DISEASES_OF_PROGRAMMED_CELL_DEATH	42	0.54594	1.85466	0.00059	0.01981	0.01677
BIOCARTA_TNFR2_PATHWAY	16	0.69084	1.85322	0.00192	0.04020	0.03404
REACTOME_LEISHMANIA_INFECTION	246	0.35645	1.61189	0.00009	0.00554	0.00470
PID_PDGFRB_PATHWAY	128	0.38645	1.61151	0.00108	0.02637	0.02233
KEGG_NITROGEN_METABOLISM	23	-0.71220	-2.01501	0.00021	0.01031	0.00873
REACTOME_ANTIGEN_ACTIVATES_B_CELL_RECEPTOR_BCR_LEADING_TO_GENERATION_OF_SECOND_MESSENGERS	32	-0.66984	-2.05903	0.00004	0.00335	0.00284
BIOCARTA_AHSP_PATHWAY	13	-0.85646	-2.10829	0.00001	0.00100	0.00085
BIOCARTA_TOB1_PATHWAY	19	-0.79523	-2.20304	0.00001	0.00095	0.00080

GSEA, Gene Set Enrichment Analysis; IPF, Idiopathic Pulmonary Fibrosis.

**Table 5:** GSEA enrichment analysis results of IPF dataset GSE110147.

Description	setSize	enrichmentScore	NES	pvalue	p.adjust	qvalue
REACTOME_DISEASES_OF_DNA_REPAIR	44	0.64885	2.12879	0.00000	0.00018	0.00015
REACTOME_G2_M_DNA_DAMAGE_CHECKPOINT	51	0.63467	2.12319	0.00000	0.00008	0.00007
REACTOME_CELL_CYCLE_CHECKPOINTS	232	0.51255	2.12125	0.00000	0.00000	0.00000
REACTOME_M_PHASE	315	0.48670	2.05585	0.00000	0.00000	0.00000
PID_BARD1_PATHWAY	27	0.69763	2.05314	0.00003	0.00145	0.00122
PID_ATM_PATHWAY	27	0.69268	2.03859	0.00004	0.00177	0.00149
WP_CILIARY_LANDSCAPE	196	0.49075	1.98455	0.00000	0.00000	0.00000
PID_PLK1_PATHWAY	41	0.61679	1.98238	0.00004	0.00167	0.00141
REACTOME_APOPTOTIC_EXECUTION_PHASE	43	0.60974	1.97742	0.00005	0.00200	0.00169
BIOCARTA_ATRBRCA_PATHWAY	18	0.69285	1.86668	0.00159	0.02713	0.02284
REACTOME_MRNA_SPLICING	170	0.46992	1.86656	0.00000	0.00020	0.00017
REACTOME_G2_M_CHECKPOINTS	119	0.48982	1.86193	0.00001	0.00071	0.00060
WP_NONALCOHOLIC_FATTY_LIVER_DISEASE	140	-0.47286	-2.09864	0.00000	0.00001	0.00001
REACTOME_METALLOTHIONEINS_BIND_METALS	11	-0.88818	-2.23369	0.00000	0.00017	0.00015
REACTOME_RESPONSE_TO_METAL_IONS	14	-0.84702	-2.30462	0.00000	0.00025	0.00021

GSEA, Gene Set Enrichment Analysis; IPF, Idiopathic Pulmonary Fibrosis.

### 3.4 Analysis of the GSE70866 dataset using WGCNA

**Figure 7:**
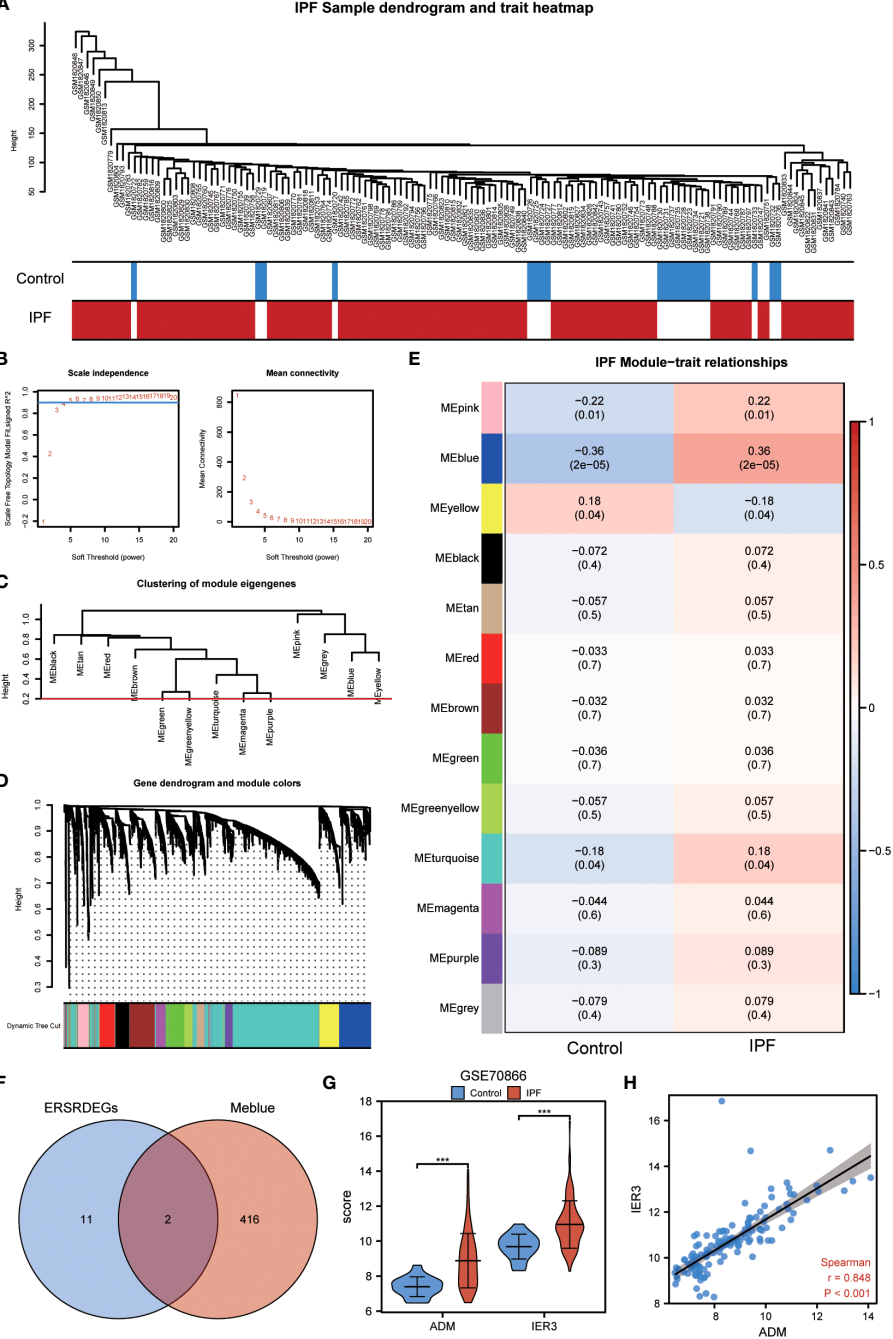
WGCNA analysis identified the coexpression modules in GSE70866 dataset. **(A)** Sample dendrogram of GSE70866 dataset. **(B)** Scale-free index analysis for soft-threshold power and mean connectivity analysis for various soft-threshold powers. **(C)** Cluster of gene modules in GSE70866 dataset. **(D)** Module clustering dendrogram based on a dissimilarity measure (1-TOM). Each color represents one module. **(E)** Heatmap of the correlation between module eigengenes and IPF, each containing the corresponding correlation and *P*-value. **(F)** Venn diagram showing overlapping genes between ERSRDEGs and genes in MEblue. **(G)** Violin plot showing the differential expression analysis of ADM and IER3 between patients with IPF and healthy individuals in the GSE70866 dataset. **(H)** Scatter plot of the correlation between ADM and IRE3. WGCNA, Weighted gene coexpression network analysis; ERSRDEGs, endoplasmic reticulum stress-related differentially expressed genes; TOM, topological overlap matrix; IPF, idiopathic pulmonary fibrosis. ***p<0.001.

The WGCNA algorithm was used to construct the coexpression modules in the GSE70866 dataset. Initially, genes exhibiting variance in the top 20% of all genes were selected as input genes. Subsequently, a hierarchical clustering analysis was employed to filter out discrete cases (**Figure 7A**). Furthermore, a soft threshold power of 5 was set as the key parameter to ensure overall connectivity of the coexpression module (**Figure 7B**). Subsequently, 13 modules were identified based on the optimal soft threshold capability, as illustrated in the cluster dendrogram (**Figure 7C**). The module merge cut height was then set to 0.2 (**Figure 7C**), resulting in the final acquisition of 13 coexpression modules with the gene clusters color-coded, as displayed in **Figure 7D**. The correlation between module membership and IPF samples is shown (**Figure 7E**). Notably, the blue module exhibited the most significant correlation with IPF (|COR|=0.36, P=2e-05) (**Figure 7E**), and its characteristic genes with the highest correlation intersected with the 13 ERSRDEGs. The Venn diagram in **Figure 7F** revealed that two genes (ADM and IER3) were common to both sets. The expression levels of two module ERSRDEGs, ADM and IER3, were analyzed using the Wilcoxon rank-sum test in the GSE70866 dataset. The results indicated that both genes were highly expressed in patients with IPF compared with normal controls (p< 0.001) (**Figure 7G**). Moreover, a significant positive correlation between the expression of the two module ERSRDEGs was observed using the Spearman algorithm (r = 0.848, p< 0.001) (**Figure 7H**).

### 3.5 Construction of ERSRDEGs-related diagnostic prediction model

**Figure 8:**
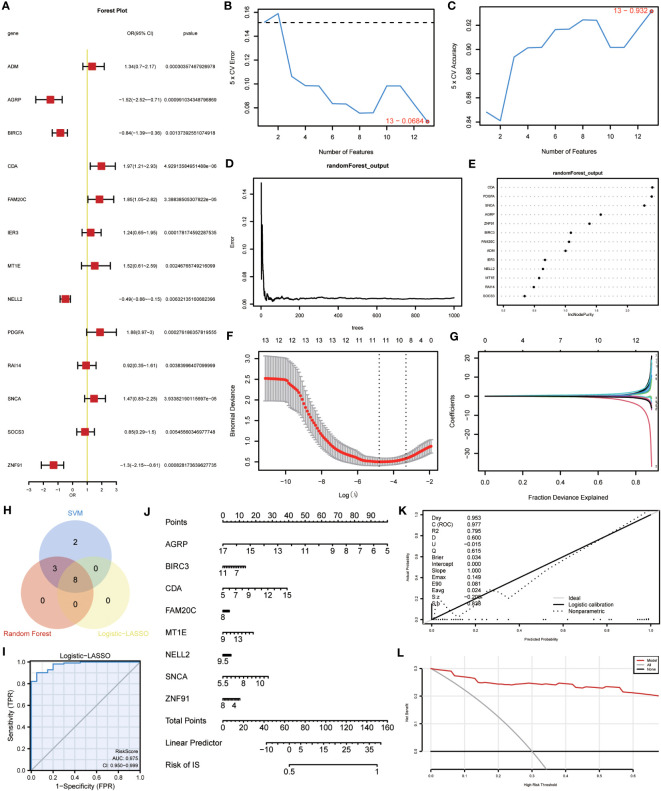
Construction of the ERSRDEGs-associated diagnostic model. **(A)** Forest plot of logistic regression model. **(B)** The number of genes with the most minimal error rate obtained by the SVM algorithm. **(C)**The number of genes with the highest accuracy obtained by the SVM algorithm. **(D)** Correlation between the error and number of trees. **(E)** The importance scores of 13 ERSRDEGs were calculated based on the RF model. **(F, G)** Screening of characteristic genes by LASSO regression analysis. **(H)** Venn diagram illustrating overlapping genes among characteristic genes selected in SVM, RF, and Logistic-LASSO models. **(I)** Nomogram predicting the ROC of prevalence in the GSE70866 series. **(J)** Nomogram of predicted prevalence according to gene score of 8 common ERSRDEGs. **(K)** Calibration curves to assess the predictive power of the diagnostic model. **(L)** DCA curve to evaluate the clinical value of the diagnostic model. SVM, support vector machine; ERSRDEGs, endoplasmic reticulum stress-related differentially expressed genes; LASSO, least absolute shrinkage, and selection operator; ROC, receiver operating characteristic curve; AUC, area under the curve; DCA, decision curve analysis.

A binary logistic regression analysis was conducted to evaluate the diagnostic significance of the 13 DEGs (ERSRDEGs) in the GSE70866 dataset for IPF. Subsequently, a logistic regression model was constructed, and the results were visually represented using forest plots (**Figure 8A**). Of the 13 genes, nine had odds ratios (OR) greater than 1, and four had OR less than 1, with statistical significance at p-value<0.05.

To further narrow down the ERSRDEGs, SVM and RF models were successively developed. The SVM model was used to determine the optimal number of genes yielding the lowest error and highest accuracy rates. The results showed that the SVM model achieved the highest accuracy when all 13 genes were used (**Figures 8B, C**).

In addition, RF analysis was used to rank and screen the most important diagnostic markers based on the expression levels of ERSRDEGs in the GSE70866 dataset. ADM, AGRP, BIRC3, CDA, FAM20C, IER3, MT1E, NELL2, PDGFA, SNCA, and ZNF91 were the top 11 genes identified (**Figures 8D, E**).

Furthermore, LASSO regression was used to identify 13 ERSRDEGs based on a previous logistic regression model with the highest predictive value for IPF (**Figure 8F, G**). Among these genes, AGRP, BIRC3, CDA, FAM20C, MT1E, NELL2, SNCA, and ZNF91 were of high importance; thus, an 8-gene signature consisting of these ERSRDEGs was constructed. The risk score was calculated as follows:


$$\begin{array}{c} RiskScore\;=AGRP*-0.417+BIRC3*-0.296+CDA*0.426\\ +FAM20C*0.418+MT1E*0.086+NELL2*-0.17\\ +SNCA*0.731+ZNF91*-0.198 \end{array}$$

To identify more reliable diagnostic markers for ERS-related conditions, we conducted an analysis to obtain co-ERSRDEGs. The results from three models, Logistic-LASSO regression, SVM, and RF, were combined. Eight co-ERSRDEGs were identified, which were also the eight ERSRDEGs obtained from the Logistic-LASSO regression model (**Figure 8H**). A diagnostic nomogram model was then established based on the expression of these genes, and the expression of AGRP was found to have a significantly higher effect on the diagnostic model than the other variables (**Figure 8J**). The accuracy of the diagnostic model was assessed using a ROC curve, which showed an AUC of 0.975 (95% CI: 0.950-0.999) for the GSE70866 dataset, indicating high prediction accuracy (**Figure 8I**). The calibration curve demonstrated that the model had good predictive performance (**Figure 8K**). Additionally, DCA was conducted to evaluate the relationship between the nomogram and gene score in predicting the benefits and risks of different cutoff points in the prevalence model for IPF. The results showed that the ERSRDEG-related diagnostic model was more beneficial at various threshold probabilities (**Figure 8L**).

### 3.6 Validation of the effect of the diagnostic prediction model and the expression of co-ERSRDEGs

**Figure 9:**
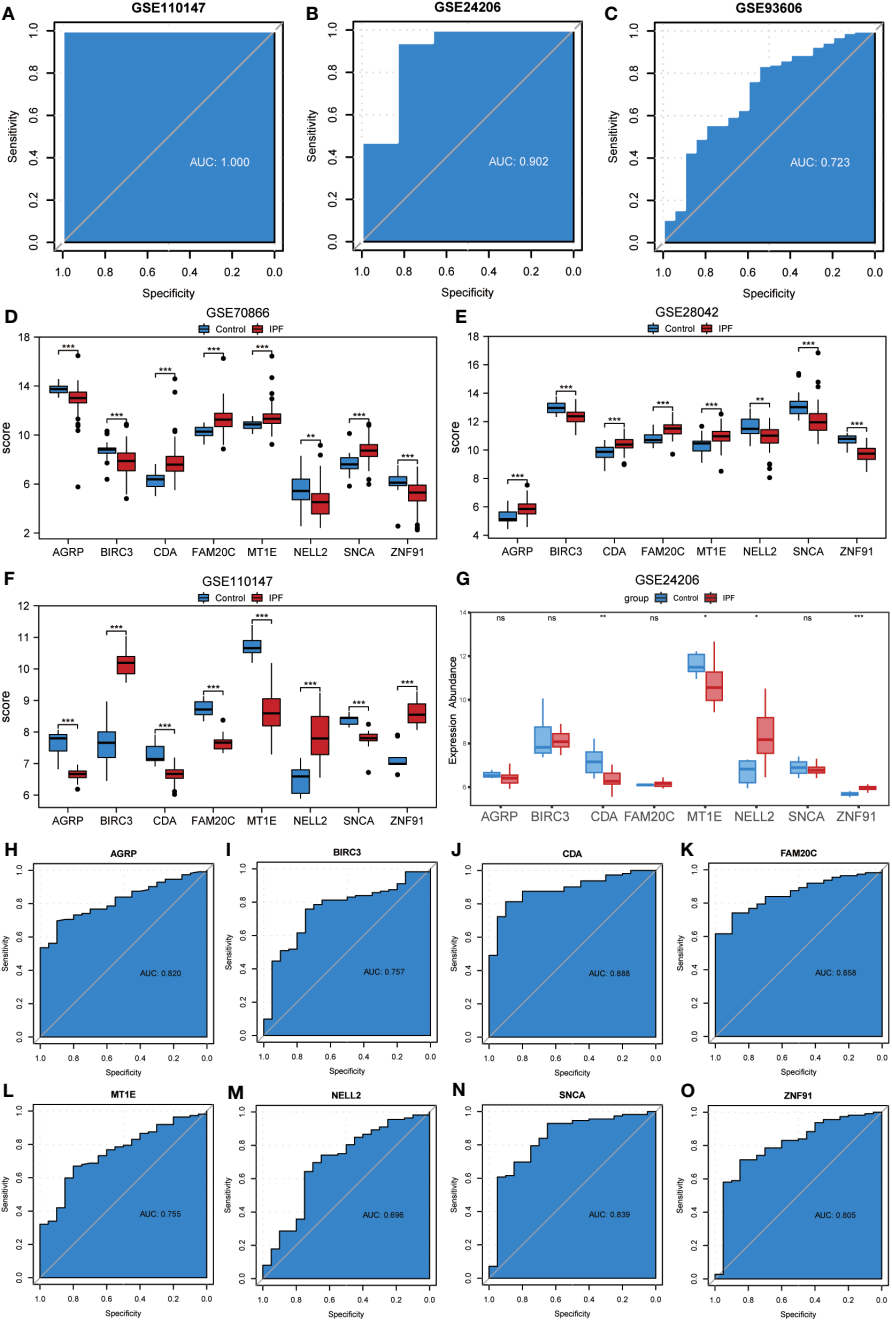
Validation of ERSRDEGs-associated diagnostic model and differential expression analysis of common ERSRDEGs. **(A-C)** Nomogram predicting the ROC of prevalence in the GSE110147 series **(A)**, GSE24206 series**(B)**, and GSE93606 series **(C)**. **(D–G)** The differential expression analysis of eight common ERSRDEGs between patients with IPF and healthy individuals in GSE70866 **(D)**, GSE28042 **(E)**, GSE110147 **(F)**, and GSE24206 series **(G)**. **(H-O)** The diagnostic efficacy of model key genes in dataset GSE70866, **(H)**AGRP, **(I)**BIRC3, **(J)**CDA, **(K)**FAM20C, **(L)** MT1E, **(M)** NELL2, **(N)** SNCA, **(O)**ZNF91. “ns,” not significant (p-value >0.05). *p<0.05, **p<0.01, ***p<0.001. IPF, idiopathic pulmonary fibrosis; ERSRDEGs, endoplasmic reticulum stress-related differentially expressed genes; ROC, receiver operating characteristic curve; AUC, area under the curve.

To assess the validity of the ERSRDEG-related diagnostic model based on the GSE70866 dataset, we evaluated the expression of eight co-ERSRDEGs in the GSE110147 dataset. Furthermore, the coefficients of these genes in the diagnostic model were used to compute the risk score for each sample in the GSE110147 dataset. Using the RiskScore and grouping information available in the GSE110147 dataset, we constructed ROC curves and observed an AUC of 1.000, indicating a high level of prediction accuracy (**Figure 9A**). To further validate our prediction method, we applied it to external datasets, specifically GSE24206 and GSE93606. The diagnostic model demonstrated a high level of prediction accuracy in the GSE24206 dataset (AUC=0.902) and relatively accurate prediction in the GSE93606 dataset (AUC=0.723) (**Figures 9B, C**). In summary, these findings suggest that our diagnostic prediction model holds significant value for BALF, PBMC, and lung tissues.

The Wilcoxon rank-sum test was used to investigate variations in the expression levels of the eight co-ERSRDEGs between patients with IPF and healthy individuals. This analysis included three training datasets (GSE70866, GSE28042, and GSE110147) and an external validation dataset (GSE28042). The results indicated that within the training set, the expression of co-ERSRDEGs was significantly different (p< 0.01) (**Figures 9D, E, H**). In the GSE24206 dataset, CDA, MT1E, NELL2, and ZNF91 expression levels also exhibited significant differences (p< 0.05). However, differences in the expression of AGRP, BIRC3, FAM20C, and SNCA were not significant (**Figure 9G**). To evaluate the diagnostic impact of the differences in the expression levels of co-ERSRDEGs on IPF, ROC curves were constructed within the GSE70866 dataset. ROC analysis revealed that, except for NELL2, all co-ERSRDEGs had AUC values exceeding 0.750 (**Figures 9H–O**).

### 3.7 Correlation analysis of expression of co-ERSRDEGs

**Figure 10:**
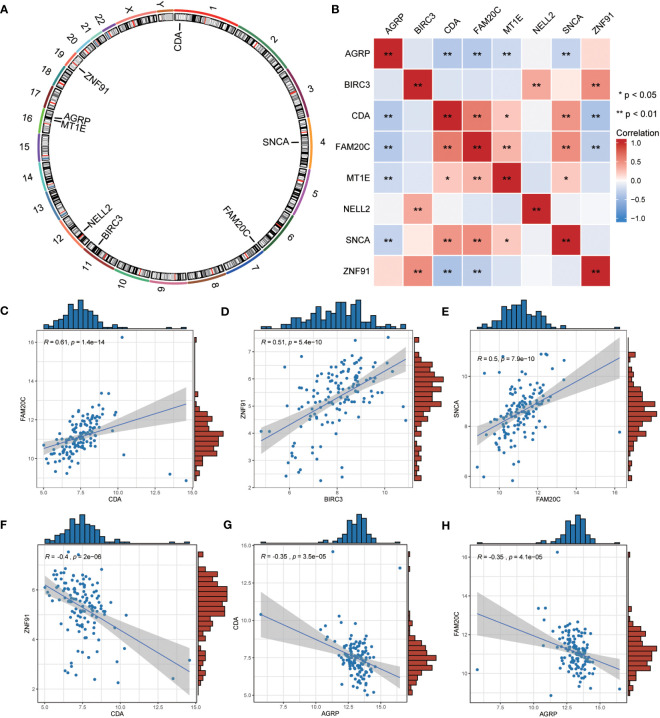
Correlation analysis among the expression of common ERSRDEGs. **(A)** The chromosomal mapping of eight common ERSRDEGs. **(B)** Correlation heatmap of eight common ERSRDEGs. **(C–H)** Scatter plot of the correlation between CDA and FAM20C **(C)**, BIRC3 and ZNF91 **(D)**, FAM20 and SNCA **(E)**, CDA and ZNF91 **(F)**, AGRP and CDA **(G)**, AGRP and FAM20C **(H)**. *p<0.05, **p<0.01, ***p<0.001. ERSRDEGs, endoplasmic reticulum stress-related differentially expressed genes.

We employed the “RCircos” package (version 1.2.2) to annotate the chromosomal positions of eight co-ERSRDEGs. Results showed that these genes were predominantly located on chromosomes 1, 4, 11, 16, and 19, with chromosome 16 exhibiting the highest distribution (**Figure 10A**). This suggests a close genomic relationship between these co-ERSRDEGs, which are in close proximity to each other on the chromosomes.

To further investigate the correlation between these eight co-ERSRDEGs in the GSE70866 dataset, we utilized the Spearman algorithm to perform a correlation analysis of their expression levels (**Figure 10B**). The results were visualized using a correlation heatmap, demonstrating that most correlations between these genes were significant (p<0.05), encompassing positive and negative associations. Subsequently, we selected the six gene pairs with the most significant positive or negative correlations and depicted them using correlation scatter plots. Notably, CDA exhibited the most significant positive correlation with FAM20C, BIRC3 with ZNF91, and FAM20C with SNCA (**Figures 10C–E**). Conversely, the most significant negative correlations were observed between CDA and ZNF91, AGRP and CDA, and AGRP and FAM20C (**Figures 10F–H**).

### 3.8 Immune infiltration in IPF dataset GSE70866 and correlation between co-ERSRDEGs and immune cells among different risk score groups

After identifying the group of patients with IPF from the GSE70866 dataset, they were further divided into high- and low-risk groups based on the median risk score obtained from the ERSRDEG-related diagnosis model. The ssGSEA and CIBERSORT algorithms were used to assess the differences in immune cell infiltration levels between the high- and low-risk-score groups.

**Figure 11:**
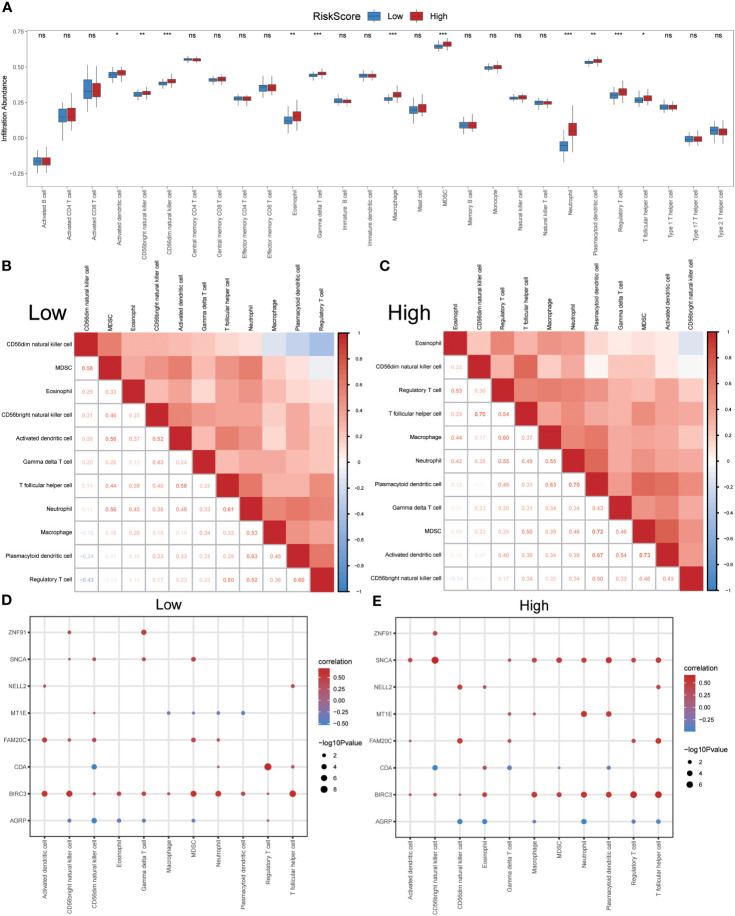
Immune infiltration analysis evaluated between groups with high- and low-risk scores by the ssGSEA algorithm in GSE70866 dataset. **(A)** The difference in expression of 28 immune cells between the high- and low-risk score groups. **(B, C)** Correlation heatmap showed the correlation coefficient between different immune cells in low- **(B)** and high-risk score groups **(C)**. **(D, E)** The correlation analysis between common ERSRDEGs and specific immune cells in the low- **(D)** and high-risk score groups **(E)**. “ns” not significant (p-value >0.05). *p<0.05, **p<0.01, ***p<0.001. ssGSEA, single-sample gene-set enrichment Analysis; ERSRDEGs, endoplasmic reticulum stress-related differentially expressed genes.

Initially, the ssGSEA algorithm was employed to calculate the abundances of 28 different types of immune cells in both groups. The Mann–Whitney U test was used to analyze differences in infiltration between the two groups. The results indicated that 11 immune cell types showed significant differences in infiltration abundance between the high- and low-risk groups (p<0.05) (**Figure 11A**). These immune cells included activated dendritic cells, CD56bright and CD56dim natural killer (NK) cells, eosinophils, gamma delta T cells, macrophages, MDSCs, neutrophils, plasmacytoid dendritic cells, regulatory T cells, and T-follicular helper cells. Furthermore, the Pearson algorithm was employed to examine the correlation between the infiltration abundances of these 11 immune cell types in both groups. The findings revealed predominantly positive correlations between the infiltration abundances of these immune cells (**Figures 11B, C**). Notably, the low-risk group exhibited the strongest positive correlation between plasmacytoid dendritic cells and neutrophils, whereas the high-risk group demonstrated the strongest positive correlation between activated dendritic cells and MDSCs. Additionally, the Pearson algorithm was used to analyze the correlation between the abundance of immune cell infiltration and the expression levels of eight co-ERSRDEGs in both groups. The results showed a correlation between the abundance of immune cell infiltration and expression levels of co-ERSRDEGs. In the low-risk group, most immune cells showed a positive association with the expression levels of co-ERSRDEGs, except for AGRP and MT1E (**Figure 11D**). In the high-risk group, most immune cells exhibited a positive correlation with the expression levels of co-ERSRDEGs, whereas only AGRP and CDA expression levels negatively correlated with the abundance of immune cell infiltration (**Figure 11E**).

**Figure 12:**
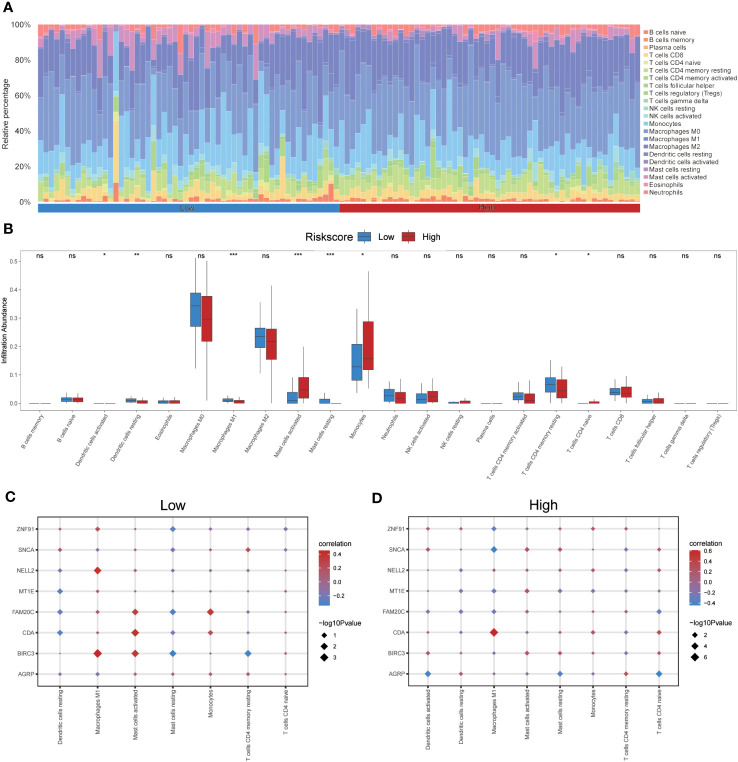
Immune infiltration analysis evaluated between groups with high- and low-risk scores by the CIBERSORT algorithm in GSE70866 dataset. **(A)** Histogram showing the distribution of 22 immune cell infiltration between the high- and low-risk score groups. **(B)** Boxplot showing the differences in infiltrated immune cells between the high- and low-risk score groups. **(C, D)** The correlation analysis between common ERSRDEGs and specific immune cells in the low- **(C)** and high-risk score groups **(D)**. “ns” not significant (p-value >0.05). *p<0.05, **p<0.01, ***p<0.001. ERSRDEGs, endoplasmic reticulum stress-related differentially expressed genes.

The CIBERSORT algorithm was used to calculate the infiltration abundance of 22 different types of immune cells in both groups. The results indicated that the infiltrating abundance of these immune cells was not uniformly zero, with macrophages M0 and M1 showing a substantial proportion of infiltration abundance across various samples (**Figure 12A**). The Wilcoxon rank-sum test was employed to examine the differences in the infiltration abundance of the 22 immune cell types between the high- and low-risk groups. The results revealed that the eight immune cell types exhibited significant differences between the two groups (p<0.05) (**Figure 12B**). These eight immune cell types include activated dendritic cells, resting dendritic cells, macrophages M1, activated mast cells, resting mast cells, monocytes, resting memory CD4 T cells, and naïve CD4 T cells. Furthermore, the Pearson algorithm was used to calculate the correlation between the abundance of these eight immune cell infiltrations and the expression levels of the eight co-ERSRDEGs in both groups. The results demonstrated a significant correlation in both groups, with the positive correlation being more pronounced than the negative correlation (**Figures 12C, D**).

### 3.9 Identification of ERS score and prognostic prediction model

**Figure 13:**
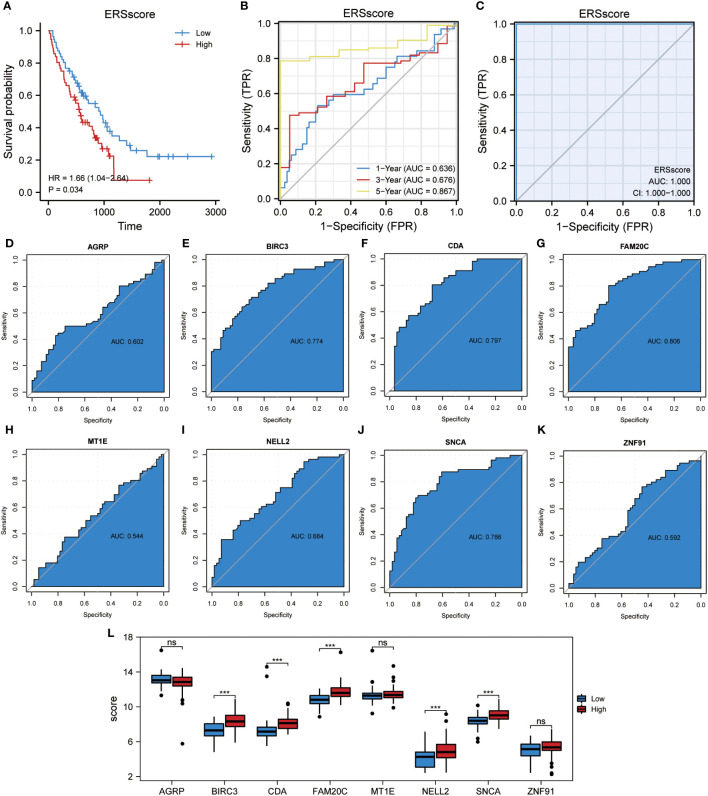
Construction of the ERS score prognostic model. **(A)** KM plot of overall survival between the high- and low-ERS score groups in the GSE70866 dataset. **(B)** ROC curve evaluated the predictive value of the model for the prognosis of patients in the discovery cohort for 1-,3-, and 5-year. **(C)** ROC curve evaluated the predictive value of the model for the prognosis of patients in the discovery cohort. **(D–K)** ROC curves of eight common ERSRDEGs in the discovery cohort, **(D)** AGRP, **(E)** BIRC3, **(F)** CDA, **(G)** FAM20C, **(H)** MT1E, **(I)** NELL2, **(J)** SNCA, **(K)** ZNF91. **(L)** The differential expression analysis of eight common ERSRDEGs between high- and low-ERS score groups. “ns” not significant (p-value >0.05). ***p<0.001. KM, Kaplan–Meier; ERS score, endoplasmic reticulum stress score; ROC, receiver operating characteristic curve; AUC, area under the curve; ERSRDEGs, endoplasmic reticulum stress-related differentially expressed genes.

In this study, the ssGSEA algorithm was used to quantify the ERS phenotype scores of all samples in the GSE70866 dataset. This score was based on the expression matrix of eight co-ERSRDEGs. Subsequently, patients with IPF were categorized into two groups, high-ERS and low-ERS, using the median ERS phenotype score as a threshold. To evaluate the prognostic value of the ERS score model in predicting the outcomes of patients with IPF, Kaplan–Meier curves were generated. The findings revealed that patients in the high ERS score group had significantly shorter OS than those in the low ERS score group (hazard ratio [HR] = 1.66, p = 0.034) (**Figure 13A**). We also employed a ROC curve to demonstrate the efficacy of the ERS score model in predicting IPF prognosis, with an AUC of 1.000 (**Figure 13C**). Additionally, a time-dependent ROC curve analysis was performed, yielding AUC values of 0.636, 0.676, and 0.867 for the 1-, 3-, and 5-year survival rates, respectively (**Figure 13B**). Furthermore, ROC curves were constructed for individual co-ERSRDEGs within the GSE70866 dataset to assess their prognostic impact on IPF. The analysis revealed that except AGRP, MT1E, NELL2, and ZNF91, the ERSRDEGs (BIRC3, CDA, FAM20C, and SNCA) exhibited AUC values exceeding 0.750 (**Figures 13D–K**). Moreover, the expression levels of certain ERSRDEGs, including BIRC3, CDA, FAM20C, NELL2, and SNCA, were higher in the high ERS score group than in the low ERS score group (**Figure 13L**). These findings suggest that the ERS score model based on co-ERSRDEGs is a promising prognostic tool for patients with IPF.

### 3.10 Immune infiltration in IPF dataset GSE70866 and correlation between co-ERSRDEGs and immune cells among different ERS score groups

Two algorithms, ssGSEA and CIBERSORT, were utilized to investigate the variations in the levels of immune cell infiltration between the high and low ERS score groups.

**Figure 14:**
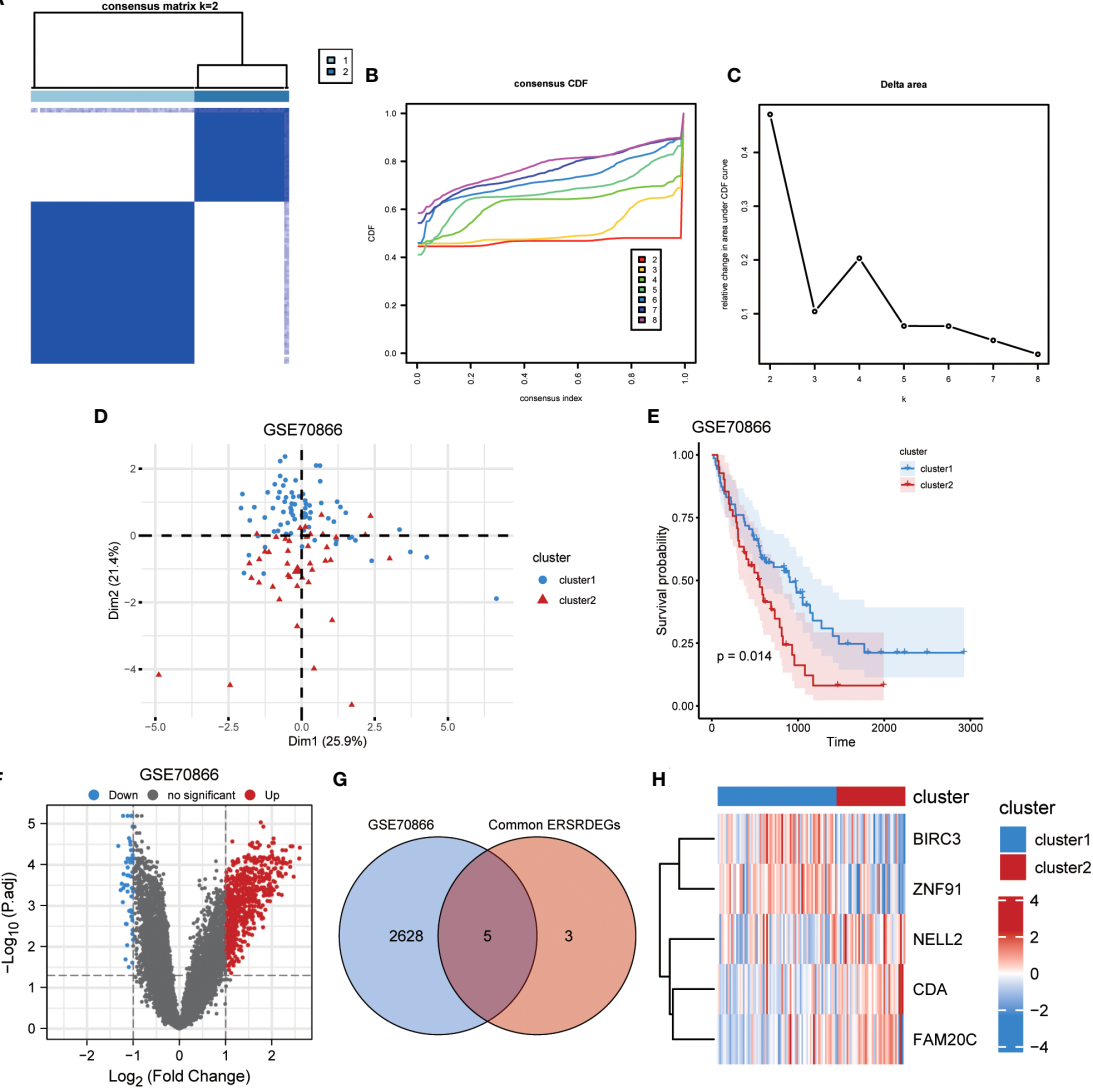
Identification of IPF subtypes. **(A)** Consensus clustering matrix when k = 2. **(B)** Consensus CDF curves when k = 2 to 8. **(C)** Relative alterations in CDF delta area curves. **(D)** PCA diagram separated clusters 1 (blue) and 2 (red) samples. **(E)** KM plot of overall survival between clusters 1 and 2 in GSE70866. **(F)** Volcano plot of differentially expressed genes between clusters 1 and 2 in GSE70866. **(G)** Venn diagram showing overlapping genes between common ERSRDEGs and DEGs between clusters 1 and 2. **(H)** Clustering heatmap of five overlapping ERSRDEGs between clusters 1 and 2 in the GSE70866 dataset. IPF, idiopathic pulmonary fibrosis; CDF, cumulative distribution function; PCA, principal component analysis; KM, Kaplan–Meier; ERSRDEGs, endoplasmic reticulum stress-related differentially expressed genes; DEGs, differentially expressed genes.

Initially, the ssGSEA algorithm was used to determine the abundance of 28 distinct types of immune cells in both groups. Subsequently, the Mann–Whitney U test was used to analyze the disparities in infiltration between the two groups. The findings revealed that 20 immune cell types exhibited significant differences in infiltration abundance between the high and low ERS score groups (p<0.05) (**Supplementary Figure 1A**). These immune cells included activated B cells, activated CD4 T cells, activated CD8 T cells, activated dendritic cells, CD56bright NK cells, CD56dim NK cells, central memory CD4 T cells, effector memory CD8 T cells, eosinophils, gamma delta T cells, immature B cells, macrophages, MDSC, monocytes, NK cells, NK T cells, neutrophils, plasmacytoid dendritic cells, regulatory T cells, T follicular helper cells, and type 1 T helper cells. Furthermore, the Pearson algorithm was employed to examine the correlation between the infiltration abundances of these 20 immune cell types in both groups. The results indicated predominantly positive correlations between the infiltration abundances of these immune cells (**Figures 14B, C**). Notably, the highest positive correlation was observed between activated CD8 T cells and effector memory CD8 + T cells in both groups. Additionally, the Pearson algorithm was used to analyze the correlation between the abundance of immune cell infiltration and the expression levels of eight common ERSRDEGs in both groups. In the low ERS score group, most immune cells exhibited a positive association with the expression levels of co-ERSRDEGs, except for AGRP (**Supplementary Figure 1D**). In the high ERS score group, most immune cells positively correlated with the expression levels of co-ERSRDEGs (**Supplementary Figure 1E**).

Concurrently, the CIBERSORT algorithm was used to calculate the infiltration abundance of 22 different types of immune cells in both groups. The results indicated that the infiltrating abundance of these immune cells was not uniformly zero, with macrophages M0 and M1 exhibiting a substantial proportion of infiltration abundance across various samples (**Supplementary Figure 2A**). The Wilcoxon rank-sum test was used to examine the differences in the infiltration abundance of the 22 immune cell types between the high and low ERS score groups. The results revealed that the nine immune cell types exhibited significant differences between the two groups (p<0.05) (**Supplementary Figure 2B**). These nine immune cell types include resting dendritic cells, macrophages M0, activated mast cells, resting mast cells, monocytes, activated NK cells, resting NK cells, resting memory CD4 T cells, and gamma delta T cells. The Pearson algorithm was used to calculate the correlation between the abundance of these nine immune cell infiltrations and the expression levels of the eight co-ERSRDEGs in both groups. The results showed that there was a relatively stronger negative correlation between immune cells and co-ERSRDEGs in the low ERS score group, whereas there was a stronger positive correlation between immune cells and co-ERSRDEGs in the high ERS score group (**Supplementary Figure 2C, D**).

### 3.11 Classification of IPF subtypes in IPF dataset GSE70866

To illustrate the ERS-related patterns of IPF, unsupervised cluster analysis was conducted on 112 IPF samples from the GSE70866 dataset. This analysis utilized the “ConsensusClusterPlus” R package and focused on the expression patterns of eight co-ERSRDEGs. In the consistency matrix of cluster 2, the intragroup correlation was higher, and the intergroup correlation was low (**Figure 14A**). Compared with clusters 2–8, the growth rate in cluster 2 was flat in the CDF plot (**Figure 14B**). Furthermore, **Figure 14C** shows a significant increase in the relative change in the area under the CDF curve from k = 2 to k = 5. Based on these findings, the 112 IPF samples were divided into two subtypes, cluster 1 (n = 71) and cluster 2 (n = 41), using PCA (**Figure 14D**). Kaplan–Meier curves were generated to evaluate the prognostic implications of disease classification. The analysis revealed that patients in cluster 2 had a significantly shorter OS than those in cluster 1 (p=0.014) (**Figure 14E**). Further analysis revealed significant differences in the gene expression patterns between the two subtypes (**Figure 14F**). We then intersected the 2,633 genes from the difference analysis with the eight co-ERSRDEG to obtain five DEGs: BIRC3, CDA, FAM20C, NELL2, and ZNF91(**Figures 14G**). To better understand the molecular characteristics that distinguish these subtypes, the expression levels of the five DEGs were evaluated. The results indicated that CDA, FAM20C, and NELL2 were significantly upregulated in cluster 2, whereas BIRC3 and ZNF91 were significantly upregulated in cluster 1 (**Figure 14H**).

### 3.12 Immune infiltration in IPF dataset GSE70866 and correlation between co-ERSRDEGs and immune cells among different IPF subtype groups

To investigate the disparities in the levels of immune cell infiltration between the two subtypes of IPF, two algorithms, ssGSEA and CIBERSORT, were utilized. Initially, the ssGSEA algorithm was used to determine the abundance of 28 distinct immune cell types in both IPF subtypes. Subsequently, the Mann–Whitney U test was used to analyze the differences in infiltration between the two subtypes. The results revealed that the five immune cell types exhibited significant differences in infiltration abundance between the two IPF subtypes (p<0.05) (**Supplementary Figure 3A**). These immune cell types included activated CD4 T cells, effector memory CD4 + T cells, immature B cells, memory B cells, and type 2 T helper cells. Furthermore, the Pearson algorithm was used to examine the correlation between the infiltration abundance of these five immune cell types in the two subtypes. The findings indicated predominantly positive correlations between the infiltration abundances of these immune cells (**Supplementary Figure 3B, C**). Notably, cluster 1 displayed the highest positive correlation between activated CD4 T cells and type 2 T helper cells, whereas cluster 2 demonstrated the highest positive correlation between effector memory CD4 T cells and memory B cells. Additionally, the Pearson algorithm was used to analyze the correlation between the abundance of immune cell infiltration and the expression levels of five DEGs (BIRC3, CDA, FAM20C, NELL2, and ZNF91) in the two subtypes. In cluster 1, most immune cells were negatively associated with the expression levels of the five DEGs (**Supplementary Figure 3D**). In cluster 2, most immune cells positively correlated with the expression levels of five DEGs, except for CDA (**Supplementary Figure 3E**).

Concurrently, the CIBERSORT algorithm was used to calculate the infiltration abundance of 22 distinct immune cell types in the two subtypes. The results indicated that the infiltrating abundance of these immune cells was not uniformly zero, with macrophages M0 and M1 exhibiting a substantial proportion of infiltration abundance across various samples (**Supplementary Figure 4A**). Furthermore, the Pearson algorithm was used to examine the correlation between the infiltration abundance of these 22 immune cell types in the two subtypes. The findings revealed that the number of positively and negatively correlated cell pairs between immune cells was approximately equal between the two subtypes (**Supplementary Figures 4B, C**). Additionally, the Pearson algorithm was used to calculate the correlation between the abundance of 22 immune cell infiltrations and the expression levels of the five DEGs in the two subtypes. The results demonstrated several positive correlations between immune cells and the five DEGs in the two subtypes (**Supplementary Figures 4D, E**).

### 3.13 Validation of expression of co-ERSRDEGs *in vivo* and *in vitro*

**Figure 15:**
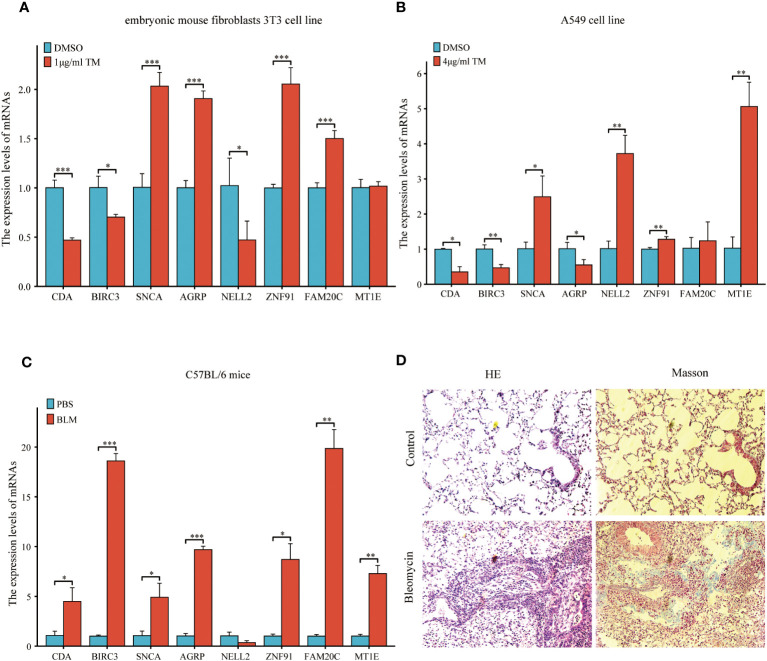
Expression of eight common ERSRDEGs genes verified via RT-qPCR in A549 cells, embryonic mouse fibroblasts 3T3 cells, and bleomycin-induced pulmonary fibrosis in mice. The experimental groups included DMSO and treatment groups *in vitro*. The treatment group was exposed to 1 μg/mL tunicamycin for 24 h in embryonic mouse fibroblasts 3T3 cells and 4 μg/mL tunicamycin for 48 h in A549 cells. The experimental group was induced with bleomycin (5 mg/kg), whereas the control group was treated with PBS *in vivo*. After 21 days, lung tissues were collected for RT-qPCR, HE, and Masson staining. **(A, B)** The expression of eight common ERSRDEGs between tunicamycin treatment and DMSO groups in embryonic mouse fibroblasts 3T3 **(A)** and A549 **(B)** cells. **(C)** The expression of eight common ERSRDEGs between bleomycin treatment and PBS groups in mice. **(D)** HE and Masson staining results of bleomycin treatment and PBS groups. *p<0.05, **p<0.01, ***p<0.001. DMSO, dimethyl sulfoxide; RT-qPCR, real-time quantitative PCR; ERSRDEGs, endoplasmic reticulum stress-related differentially expressed genes.

A previous analysis demonstrated a strong correlation between ERS and the diagnosis, prognosis, and classification of IPF. To validate the mRNA expression of the eight co-ERSRDEGs, we established A549 cell and embryonic mouse fibroblast 3T3 cell ERS models using tunicamycin. RT-qPCR results revealed that SNCA, AGRP, ZNF91, FAM20C, and MT1E exhibited high expression levels, whereas CDA, BIRC3, and NELL2 were relatively low in the tunicamycin-treated group compared with the control group in embryonic mouse fibroblast 3T3 cells. This difference was significant, except for MT1E (**Figure 15A**). Similarly, in the A549 cell line, RT-qPCR results indicated high SNCA, NELL2, ZNF91, MT1E, and FAM20C expression levels. In contrast, CDA, BIRC3, and AGRP levels were relatively lower in the tunicamycin-treated group than in the control group. The difference was significant, except for FAM20C (**Figure 15B**).

To further validate the findings of the *in vitro* cell experiments and simulate the pathological process of pulmonary fibrosis in patients, we successfully established a bleomycin-induced pulmonary fibrosis mouse model for *in vivo* experiments. This model allowed us to investigate ERS events during the development of pulmonary fibrosis. RT-qPCR results demonstrated significantly high expression levels of CDA, BIRC3, SNCA, AGRP, ZNF91, FAM20C, and MT1E in the lung tissues of mice treated with bleomycin. However, results showed that NELL2 was weakly expressed in the bleomycin-induced lung tissues of mice. However, the difference was not significant (**Figure 15C**). Additionally, histological examination using HE and Masson staining revealed significantly increased lung tissue inflammation and collagen deposition in the bleomycin group compared with the control group (**Figure 15D**).

## 4 Discussion

IPF is a prevalent form of interstitial lung disease characterized by complex and multifaceted pathogenesis and progression (45). The initial presentation of IPF is often characterized by non-specific symptoms, leading to significant delays in diagnosis. The heterogeneity of IPF poses challenges to the effectiveness of treatment strategies, resulting in a poor prognosis for affected individuals (46). Furthermore, the prediction of the course and prognosis of IPF on an individual basis remains challenging. In recent years, numerous studies have been conducted to identify the clinical, imaging, and pathological indicators that could aid in diagnosing, predicting progression, and estimating survival in IPF (3). However, the retrospective nature of these studies and the inherent uncertainty associated with the employed metrics have resulted in varying degrees of accuracy for the identified predictors. Consequently, there is an urgent need to develop appropriate and precise diagnostic and prognostic models for IPF to improve clinical practice. ERS has been extensively investigated for its association with IPF. Although its involvement in the pathogenesis and progression of IPF is likely, the exact mechanism remains unclear.

Given that IPF is a complex pathophysiological process, this study aimed to explore the significance of ERS in the diagnosis and prognosis of IPF using samples derived from the BALF, PBMC, and lung tissue. Initially, we identified and validated eight ERSRGs to diagnose IPF: AGRP, BIRC3, CDA, FAM20C, MT1E, NELL2, SNCA, and ZNF91. Additionally, we explored the association between ERS and IPF outcomes by calculating an ERS score based on the expression levels of eight co-ERSRDEGs. These genes have been identified as potential protective prognostic factors. Consistent clustering was performed to further predict the regulatory patterns of ERS-related prognosis. This analysis revealed two subgroups with significantly different prognoses, with cluster 2 exhibiting poorer outcomes than cluster 1. Moreover, we discovered a correlation between the infiltration of various immune cell types and ERS in the lung tissue of patients with IPF, as well as differences in immune cell infiltration between normal and IPF lung tissues. Our findings indicated that ERS plays a crucial role in IPF development. Notably, this study represents the first bioinformatics investigation to elucidate the relationship between ERS and IPF using human samples and has laid the foundation for further exploration of ERS in the context of IPF. Therefore, the identification of novel therapeutic targets is imperative.

In this study, diagnostic and prognostic models comprised eight ERS-related genes. AGRP, also known as agouti-related neuropeptide, encodes an antagonist of the melanocortin-3 and melanocortin-4 receptors. Its role in regulating feeding behavior through the melanocortin receptor and/or intracellular calcium regulation suggests its involvement in weight homeostasis (47). Previous studies on body weight homeostasis have shown a correlation between AGRP and ERS and the inflammatory response. However, the specific mechanism of action may be contradictory. Zhou et al. demonstrated that antipsychotic medications, such as olanzapine, induce ERS in hypothalamic neurons, leading to increased expression of neuropeptide Y and AGRP, autophagy, and resistance to leptin and insulin (48). This ultimately results in the inflammation of the central nervous system (CNS), leading to weight gain. However, Hagimoto et al. showed that glucocorticoid-induced AGRP expression is suppressed through the NF-κB-p65 pathway in ERS (49). Currently, there is a lack of research examining the role of AGRP in the context of pulmonary fibrosis. This study showed that AGRP expression has a notably greater impact on the ERS-related diagnostic model than other co-ERSRDEGs. Furthermore, AGRP expression was negatively correlated with immune cell infiltration. This finding contradicts the positive correlation between AGRP and inflammation reported by Zhou et al., necessitating further comprehensive investigations.

BIRC3, also known as cellular IAP2, is a member of the human inhibitor of apoptosis protein (IAP) family (50). Previous research has demonstrated that BIRC3 is a multifunctional protein that regulates caspases, apoptosis, inflammatory signaling, immunity, mitogenic kinase signaling, and cell proliferation (51). In specific cell types, such as polarized human myeloid leukemia THP-1 cells and primary human macrophages, cIAP1 and cIAP2 are highly expressed in M1 macrophages (52). Studies have indicated that BIRC3 regulates immune-related lung diseases, including asthma and *Klebsiella pneumoniae* pneumonia. Increased BIRC3 expression may contribute to asthma pathogenesis by influencing eosinophilic and allergic inflammation (53). In a mouse model of infection, blocking the Birc3/TLR4/Myd88 signaling pathway showed a protective effect against carbapenem-resistant *K. pneumoniae* (54). Additionally, studies have revealed the involvement of ERS and BIRC3 in tumor regulation. SNHG1, a KLF4-regulated lncRNA, suppresses ERS-induced apoptosis and promotes gliomagenesis by increasing BIRC3 expression (55). BIRC3 may also play a role in liver fibrosis by regulating the inflammatory response. Targeting cIAPs, including BIRC3, has been suggested as a potential therapeutic strategy for liver fibrosis by increasing MMP9 expression induced by CCL5 chemotactic neutrophils (56). Notably, recent studies have proposed targeting IAPs as a potential therapy for IPF by promoting apoptosis of mesenchymal cells. Fibroblasts from IPF tissues show increased expression of XIAP, which is also a member of the IAP family and is associated with resistance to apoptosis in lung fibroblasts (57). Furthermore, research has demonstrated that the profibrotic mediator TGF-β1 could enhance the expression of both XIAP and cIAPs in murine mesenchymal cells. Increased expression of XIAP and cIAP1 has also been observed in bleomycin-induced IPF models. Consistently, the IAPs inhibitor AT-406 protected mice from bleomycin-induced lung fibrosis (58). In this study, the AUC value of BIRC3 in the diagnostic and prognostic models of IPF exceeded 0.750. Moreover, there was a positive association between the expression levels of BIRC3 and the extent of immune cell infiltration. These findings suggest that BIRC3 is important in the context of IPF, and further investigation is warranted to elucidate its underlying mechanism.

Metallothionein (MT) is a low-molecular-weight protein with a high cysteine content that binds to metals and is present in all eukaryotes. In humans, MT is categorized into four subfamilies: MT1, MT2 (also known as MT2A), MT3, and MT4 (59). MT1 is involved in ERS regulation Zn treatment prevents type 1 diabetes-induced hepatic oxidative damage, ERS, and cell death by upregulating hepatic MT expression (60). MT also protects against ERS-induced cardiac anomalies, possibly by attenuating cardiac autophagy (61). Furthermore, MT1 plays a role in the differentiation and functioning of immune cells. It positively regulates the differentiation of CD4+ T cells into Tregs and negatively regulates the differentiation of CD4+ T cells into Tr1 and Th17 cells (62). MT1 may also regulate lung diseases, such as chronic obstructive pulmonary disease (COPD), by affecting immune responses. Researchers identified a subpopulation of macrophages with high MT expression in patients with advanced COPD (63). Moreover, MT1 regulates pulmonary fibrosis, and its induction attenuates the progression of lung fibrosis in mice exposed to long-term intermittent hypoxia (64). In this study, MT1 expression in BALF and PBMC was significantly higher in patients with IPF than in healthy individuals. Conversely, the expression of MT1 in the lung tissue was lower in patients with IPF than in healthy controls. Furthermore, the AUC value of MT1 in the diagnostic model for IPF was 0.755, indicating its potential as a diagnostic marker for this condition. Interestingly, a negative association was observed between the expression levels of MT1 and extent of immune cell infiltration. This finding is in contrast to previous studies investigating the relationship between MT1 and inflammation, suggesting the need for further investigation.

Alpha-synuclein (SNCA) is a member of the synuclein protein family, which includes α-, β-, and γ-synuclein, as well as synoretin (65). It is primarily expressed in regions of the adult CNS associated with synaptic plasticity (66). SNCA and ERS have been implicated in several neurological disorders. The aggregation of SNCA disrupts the ability of neurons to respond to misfolded proteins in the ER. It has been suggested that enhancing multiple proteostatic pathways is therapeutically beneficial in Parkinson’s disease (PD) (67). Additionally, SNCA is involved in ERS induced by manganese through the PERK signaling pathway in brain slice cultures (68). SNCA and inflammation also play a role in the development and progression of neurological diseases, including PD. Intracellular translocation of toxic SNCA species could trigger hyperactivity in microglia, activate astrocytes, upregulate the expression of proinflammatory factors, and recruit peripheral immune cells to the vicinity of preapoptotic and apoptotic dopaminergic neurons in the CNS, all of which could contribute to neuronal dysfunction (69). Studies have shown that SNCA is involved in the regulation of renal fibrosis. Disruption of SNCA signaling in renal proximal tubular epithelial cells contributes to the pathogenesis of renal tubulointerstitial fibrosis by promoting a partial epithelial-to-mesenchymal transition and accumulation of ECM (70). This study showed that SNCA expression exhibited a notable increase in the high ERS score group compared with the low ERS score group among patients diagnosed with IPF. Additionally, the AUC value of SNCA in the diagnostic and prognostic models for IPF exceeded 0.750, indicating its potential as a valuable diagnostic and prognostic marker for this particular condition. Furthermore, a positive correlation was observed between SNCA expression and the extent of immune cell infiltration. Collectively, these findings suggest that SNCA plays a significant role in the development and progression of pulmonary fibrosis via ERS.

Cytidine deaminase (CDA) is an enzyme that plays a crucial role in the pyrimidine salvage pathway (71). Family with sequence similarity 20 member C (FAM20C), previously known as Golgi casein kinase (G-CK), is a protein specifically localized in the Golgi apparatus (72). NELL2 is a glycoprotein involved in the development of neural cells and guidance of axons, including their repulsion (73). Zinc finger protein 91 (ZNF91) is a nuclear protein that is 63.5 kDa in size and exhibits structural motifs characteristic of transcription factors (73). These proteins were associated with the GO enrichment analysis results of ERSRDEGs. ERSRDEGs were enriched in the negative regulation of the phosphate metabolic process and G protein-coupled receptor signaling pathway. Additionally, they were enriched in cellular components of the Golgi lumen. Regarding MF, ERSRDEGs were closely associated with enzyme inhibitors, receptor ligands, and signaling receptor activator activities.

This study showed significant differences in the expression of AGRP, BIRC3, CDA, FAM20C, MT1E, NELL2, SNCA, and ZNF91 between patients with IPF and healthy controls. These were also significantly correlated with immune cell infiltration. Furthermore, our *in vivo* and *in vitro* experiments demonstrated that the expression of these molecules differed between the tunicamycin and bleomycin groups compared with the normal control group. This suggests that these molecules are involved in the development of IPF via ERS and inflammatory responses. However, it is important to note that the inconsistent trend in their expression may be attributed to the different sources of samples analyzed in our database, including BALF, PBMC, and lung tissue. Additionally, the A549 cells used in our *in vitro* experiments represent alveolar epithelial cells, whereas the 3T3 cells represent lung fibroblasts. Therefore, AGRP, NELL2, FAM20C, and MT1E may exert varying effects on alveolar epithelial cells and lung fibroblasts. Moreover, our *in vivo* experiments used bleomycin to induce pulmonary fibrosis in mice, which may not fully reflect the pathophysiological processes of IPF in humans. Furthermore, the stress state of the ER in mice might not have been fully represented in our *in vivo* experiments without the addition of tunicamycin. An important consideration regarding the use of animal models is that they may not completely mimic the pathophysiology of IPF. Nevertheless, animal models provide detailed mechanistic insights that are difficult to obtain from human studies. In addition, we did not assess the protein levels of these molecules. Therefore, further investigation is required to explore the changes in protein levels and necessitate including more BALF, blood, and lung tissue samples from patients with IPF for verification.

Recent advances have shed light on the role of the immune system in IPF, revealing that immune dysregulation is a crucial factor in the pathophysiology of the disease (14, 15). Both human and mouse studies have provided evidence that monocytes are recruited to the lungs in response to tissue injury and subsequently differentiate into long-lived alveolar macrophages (Ams) (74). These Ams play a pivotal role in promoting fibrosis through various mechanisms, including the production of TGF-β (75), chemokine ligand 18 (CCL18) (76), chitinase 3-like protein 1 (CHI3L1) (77), matrix metalloproteinases (MMPs) (78), and activation of the Wnt/β-catenin pathway (79). These processes ultimately lead to fibroblast activation, myofibroblast differentiation, and ECM remodeling. Importantly, several of these profibrotic mechanisms are associated with M2 polarization in Ams (79). Th17 cells, CD8+ T cells, and Tregs have been observed to contribute to the progression of fibrosis, whereas Th1 cells and tissue-resident memory CD4+ T cells have shown potential protective effects (15). In accordance with previous studies, our findings demonstrated a higher abundance of monocytes and macrophages in the high-risk group than in the low-risk group, suggesting a potential association between elevated levels of these immune cells and IPF progression. Additionally, we observed a decrease in the number of resting memory CD4+ T cells in the BALF of the high-risk group, indicating the potential protective role of these cells in IPF. Furthermore, significant differences were observed in the dendritic cells, mast cells, NK cells, neutrophils, and myeloid-derived suppressor cells (MDSC) between the high- and low-risk groups. Notably, a significant correlation was observed between most ERSRDEGs and various immune cell populations. However, the precise contribution of these immune cells to IPF pathogenesis requires further investigation.

This study established a correlation between ERS and its associated genes in IPF diagnosis and progression. However, this study had some limitations. First, it relied on data from the GEO database, which may have introduced potential biases. Despite including multiple datasets, the sample size was relatively small, which may have affected the generalizability of our findings. Second, the majority of the datasets lacked crucial clinical information such as pulmonary function parameters, St. George’s respiratory questionnaire scores, and the use of antifibrotic medications. Lastly, the relationship between the risk score and immune activity requires further investigation through basic experiments. Therefore, additional research is necessary to validate the clinical significance of these results.

## Data availability statement

The datasets presented in this study can be found in online repositories. The names of the repository/repositories and accession number(s) can be found in the article/**Supplementary Material**.

## Ethics statement

The studies involving humans were approved by the Human Tissue Committees and Research Ethics Boards of the University Health Network (protocol n.11–0932) and Western University (n.105214). The studies were conducted in accordance with the local legislation and institutional requirements. Written informed consent for participation was not required from the participants or the participants’ legal guardians/next of kin in accordance with the national legislation and institutional requirements.

## Author contributions

HZ: Conceptualization, Data curation, Formal analysis, Methodology, Project administration, Software, Validation, Writing – original draft, Writing – review & editing. AZ: Formal analysis, Methodology, Writing – original draft. MZ: Investigation, Project administration, Writing – original draft. LP: Formal analysis, Writing – original draft. XW: Data curation, Writing – original draft. CF: Software, Writing – original draft. LG: Writing – original draft, Visualization. WY: Conceptualization, Investigation, Writing – original draft. DL: Conceptualization, Supervision, Validation, Writing – review & editing. YC: Conceptualization, Funding acquisition, Resources, Supervision, Writing – review & editing.

## Supplementary material

Supplementary Figure 1 Immune infiltration analysis evaluated between groups with high and low ERS scores by the ssGSEA algorithm in GSE70866 dataset. **(A)** The difference in expression of 28 immune cells between the high- and low-ERS score groups. **(B, C)** Correlation heatmap showed the correlation coefficient between different immune cells in the low-**(B)** and high-ERS**(C)** score groups. **(D, E)** The correlation analysis between common ERSRDEGs and specific immune cells in the low-**(D)** and high-ERS**(E)** score groups. “ns”, not significant (p-value >0.05). *p<0.05, **p<0.01, ***p<0.001. ssGSEA, single-sample gene-set enrichment analysis; ERS score, endoplasmic reticulum stress score; ERSRDEGs, endoplasmic reticulum stress-related differentially expressed genes.

Supplementary Figure 2 Immune infiltration analysis evaluated between groups with high and low ERS scores by the CIBERSORT algorithm in GSE70866 dataset. **(A)** Histogram showing the distribution of 22 immune cell infiltration between the high- and low-ERS score groups. **(B)** Boxplot showing the differences in infiltrated immune cells between the high- and low-ERS score groups. **(C, D)** The correlation analysis between common ERSRDEGs and specific immune cells in the low-**(C)** and high-ERS**(D)** score groups. “ns”, not significant (p-value >0.05). *p<0.05, **p<0.01, ***p<0.001. ERS score, endoplasmic reticulum stress score; ERSRDEGs, endoplasmic reticulum stress-related differentially expressed genes.

Supplementary Figure 3 Immune infiltration analysis evaluated between clusters 1 and 2 by the ssGSEA algorithm in GSE70866 dataset. **(A)** The difference in expression of 28 immune cells between clusters 1 and 2. **(B, C)** Correlation heatmap showed the correlation coefficient between different immune cells in clusters 1**(B)** and 2**(C)**. **(D, E)** The correlation analysis between common ERSRDEGs and specific immune cells in clusters 1 **(D)** and 2 **(E)**. “ns”, not significant (p-value >0.05). *p<0.05, **p<0.01, ***p<0.001. ssGSEA, single-sample gene-set enrichment analysis; ERSRDEGs, endoplasmic reticulum stress-related differentially expressed genes.

Supplementary Figure 4 Immune infiltration analysis evaluated between clusters 1 and 2 by the CIBERSORT algorithm in GSE70866 dataset. **(A)** Histogram showing the distribution of 22 immune cell infiltration between clusters 1 and 2. **(B, C)** Correlation heatmap showed the correlation coefficient between different immune cells in clusters 1**(B)** and 2**(C)**. **(D, E)** The correlation analysis between common ERSRDEGs and specific immune cells in clusters 1 **(D)** and 2 **(E)**. *p<0.05, **p<0.01, ***p<0.001. ERSRDEGs, endoplasmic reticulum stress-related differentially expressed genes.

## References

[1] Podolanczuk AJ Thomson CC Remy-Jardin M Richeldi L Martinez FJ Kolb M. Idiopathic pulmonary fibrosis: state of the art for 2023. Eur Respir J (2023) 61(4):2200957. doi: 10.1183/13993003.00957-2022 36702498

[2] Liu GY Budinger GRS Dematte JE. Advances in the management of idiopathic pulmonary fibrosis and progressive pulmonary fibrosis. BMJ (2022) 377:e066354. doi: 10.1136/bmj-2021-066354 36946547

[3] Spagnolo P Kropski JA Jones MG Lee JS Rossi G Karampitsakos T. Idiopathic pulmonary fibrosis: Disease mechanisms and drug development. Pharmacol Ther (2021) 222:107798. doi: 10.1016/j.pharmthera.2020.107798 33359599 PMC8142468

[4] Moss BJ Ryter SW Rosas IO. Pathogenic mechanisms underlying idiopathic pulmonary fibrosis. Annu Rev Pathol (2022) 17:515–46. doi: 10.1146/annurev-pathol-042320-030240 34813355

[5] Drakopanagiotakis F Wujak L Wygrecka M Markart P. Biomarkers in idiopathic pulmonary fibrosis. Matrix Biol (2018) 68–69:404–21. doi: 10.1016/j.matbio.2018.01.023 29408012

[6] Jee AS Sahhar J Youssef P Bleasel J Adelstein S Nguyen M. Review: Serum biomarkers in idiopathic pulmonary fibrosis and systemic sclerosis associated interstitial lung disease – Frontiers and horizons. Pharmacol Ther (2019) 202:40–52. doi: 10.1016/j.pharmthera.2019.05.014 31153954

[7] Marciniak SJ Chambers JE Ron D. Pharmacological targeting of endoplasmic reticulum stress in disease. Nat Rev Drug Discovery (2022) 21(2):115–40. doi: 10.1038/s41573-021-00320-3 34702991

[8] Chen X Shi C He M Xiong S Xia X. Endoplasmic reticulum stress: molecular mechanism and therapeutic targets. Signal Transduct Target Ther (2023) 8(1):352. doi: 10.1038/s41392-023-01570-w 37709773 PMC10502142

[9] Burman A Tanjore H Blackwell TS. Endoplasmic reticulum stress in pulmonary fibrosis. Matrix Biol (2018) 68–69:355–65. doi: 10.1016/j.matbio.2018.03.015 PMC6392005 29567124

[10] Borok Z Horie M Flodby P Wang H Liu Y Ganesh S. Grp78 loss in epithelial progenitors reveals an age-linked role for endoplasmic reticulum stress in pulmonary fibrosis. Am J Respir Crit Care Med (2020) 201:198–211. doi: 10.1164/rccm.201902-0451OC 31738079 PMC6961744

[11] Meng X Liu K Xie H Zhu Y Jin W Lu J. Endoplasmic reticulum stress promotes epithelial−mesenchymal transition *via* the PERK signaling pathway in paraquat−induced pulmonary fibrosis. Mol Med Rep (2021) 24(1):525. doi: 10.3892/mmr.2021.12164 34036384 PMC8170262

[12] Ghavami S Yeganeh B Zeki AA Shojaei S Kenyon NJ Ott S. Autophagy and the unfolded protein response promote profibrotic effects of TGF-β1 in human lung fibroblasts. Am J Physiol Lung Cell Mol Physiol (2018) 314:L493–504. doi: 10.1152/ajplung.00372.2017 PMC5900356 29074489

[13] Lee TH Yeh CF Lee YT Shih YC Chen YT Hung CT. Fibroblast-enriched endoplasmic reticulum protein TXNDC5 promotes pulmonary fibrosis by augmenting TGFβ signaling through TGFBR1 stabilization. Nat Commun (2020) 11:4254. doi: 10.1038/s41467-020-18047-x 32848143 PMC7449970

[14] Adams TS Schupp JC Poli S Ayaub EA Neumark N Ahangari F. Single-cell RNA-seq reveals ectopic and aberrant lung-resident cell populations in idiopathic pulmonary fibrosis. Sci Adv (2020) 6(28):eaba1983. doi: 10.1126/sciadv.aba1983 32832599 PMC7439502

[15] Shenderov K Collins SL Powell JD Horton MR. Immune dysregulation as a driver of idiopathic pulmonary fibrosis. J Clin Invest (2021) 131(2):e143226. doi: 10.1172/JCI143226 33463535 PMC7810481

[16] Ayaub EA Kolb PS Mohammed-Ali Z Tat V Murphy J Bellaye PS. GRP78 and CHOP modulate macrophage apoptosis and the development of bleomycin-induced pulmonary fibrosis. J Pathol (2016) 239:411–25. doi: 10.1002/path.4738 27135434

[17] Yao Y Wang Y Zhang Z He L Zhu J Zhang M. Chop deficiency protects mice against bleomycin-induced pulmonary fibrosis by attenuating M2 macrophage production. Mol Ther (2016) 24:915–25. doi: 10.1038/mt.2016.36 PMC4881771 26883801

[18] Barrett T Troup DB Wilhite SE Ledoux P Rudnev D Evangelista C. NCBI GEO: Mining tens of millions of expression profiles–Database and tools update. Nucleic Acids Res (2007) 35:D760–5. doi: 10.1093/nar/gkl887 PMC1669752 17099226

[19] Prasse A Binder H Schupp JC Kayser G Bargagli E Jaeger B. BAL cell gene expression is indicative of outcome and airway basal cell involvement in idiopathic pulmonary fibrosis. Am J Respir Crit Care Med (2019) 199:622–30. doi: 10.1164/rccm.201712-2551OC PMC6396865 30141961

[20] Herazo-Maya JD Noth I Duncan SR Kim S Ma SF Tseng GC. Peripheral blood mononuclear cell gene expression profiles predict poor outcome in idiopathic pulmonary fibrosis. Sci Transl Med (2013) 5:205ra136. doi: 10.1126/scitranslmed.3005964 PMC4175518 24089408

[21] Cecchini MJ Hosein K Howlett CJ Joseph M Mura M. Comprehensive gene expression profiling identifies distinct and overlapping transcriptional profiles in nonspecific interstitial pneumonia and idiopathic pulmonary fibrosis. Respir Res (2018) 19:153. doi: 10.1186/s12931-018-0857-1 30111332 PMC6094889

[22] Meltzer EB Barry WT D’Amico TA Davis RD Lin SS Onaitis MW. Bayesian probit regression model for the diagnosis of pulmonary fibrosis: Proof-of-principle. BMC Med Genomics (2011) 4:70. doi: 10.1186/1755-8794-4-70 21974901 PMC3199230

[23] Molyneaux PL Willis-Owen SAG Cox MJ James P Cowman S Loebinger M. Host-Microbial interactions in idiopathic pulmonary fibrosis. Am J Respir Crit Care Med (2017) 195:1640–50. doi: 10.1164/rccm.201607-1408OC. hosts. PMC5476909 28085486

[24] Davis S Meltzer PS. GEOquery: A bridge between the gene expression omnibus (GEO) and bioconductor. Bioinformatics (2007) 23:1846–7. doi: 10.1093/bioinformatics/btm254 17496320

[25] Stelzer G Rosen N Plaschkes I Zimmerman S Twik M Fishilevich S. The GeneCards suite: From gene data mining to disease genome sequence analyses. Curr Protoc Bioinf (2016) 54:1.30.1–1.30.33. doi: 10.1002/cpbi.5 27322403

[26] Lai Y Lin X Lin C Lin X Chen Z Zhang L. Identification of endoplasmic reticulum stress-associated genes and subtypes for prediction of Alzheimer’s disease based on interpretable machine learning. Front Pharmacol (2022) 13:975774. doi: 10.3389/fphar.2022.975774 36059957 PMC9438901

[27] Shen Y Cao Y Zhou L Wu J Mao M. Construction of an endoplasmic reticulum stress-related gene model for predicting prognosis and immune features in kidney renal clear cell carcinoma. Front Mol Biosci (2022) 9:928006. doi: 10.3389/fmolb.2022.928006 36120545 PMC9478755

[28] Ritchie ME Phipson B Wu D Hu Y Law CW Shi W. Limma powers differential expression analyses for RNA-sequencing and microarray studies. Nucleic Acids Res (2015) 43:e47. doi: 10.1093/nar/gkv007 25605792 PMC4402510

[29] Yu G Wang LG Han Y He QY. clusterProfiler: An R package for comparing biological themes among gene clusters. Omics A J Integr Biol (2012) 16:284–7. doi: 10.1089/omi.2011.0118 PMC3339379 22455463

[30] Subramanian A Tamayo P Mootha VK Mukherjee S Ebert BL Gillette MA. Gene set enrichment analysis: A knowledge-based approach for interpreting genome-wide expression profiles. Proc Natl Acad Sci U.S.A. (2005) 102:15545–50. doi: 10.1073/pnas.0506580102 PMC1239896 16199517

[31] Zhang B Horvath S. A general framework for weighted gene co-expression network analysis. Stat Appl Genet Mol Biol (2005) 4:article17. doi: 10.2202/1544-6115.1128. 16646834

[32] Langfelder P Horvath S. WGCNA: An R package for weighted correlation network analysis. BMC Bioinf (2008) 9:559. doi: 10.1186/1471-2105-9-559 PMC2631488 19114008

[33] Sanz H Valim C Vegas E Oller JM Reverter F. SVM-RFE: Selection and visualization of the most relevant features through non-linear kernels. BMC Bioinf (2018) 19:432. doi: 10.1186/s12859-018-2451-4 PMC6245920 30453885

[34] Gruber HE Hoelscher GL Ingram JA Hanley EN Jr. Genome-wide analysis of pain-, nerve- and neurotrophin -related gene expression in the degenerating human annulus. Mol Pain (2012) 8:63. doi: 10.1186/1744-8069-8-63 22963171 PMC3495673

[35] Liu Y Zhao H. Variable importance-weighted random forests. Quant Biol (2017) 5:338–51. doi: 10.1007/s40484-017-0121-6 PMC6051549 30034909

[36] Cai W van der Laan M. Nonparametric bootstrap inference for the targeted highly adaptive least absolute shrinkage and selection operator (LASSO) estimator. Int J Biostat (2020) 16. doi: 10.1515/ijb-2017-0070 32772002

[37] Engebretsen S Bohlin J. Statistical predictions with glmnet. Clin Epigenet (2019) 11:123. doi: 10.1186/s13148-019-0730-1 PMC6708235 31443682

[38] Park SY. Nomogram: An analogue tool to deliver digital knowledge. J Thorac Cardiovasc Surg (2018) 155:1793. doi: 10.1016/j.jtcvs.2017.12.107 29370910

[39] Van Calster B Wynants L Verbeek JFM Verbakel JY Christodoulou E Vickers AJ. Reporting and interpreting decision curve analysis: A guide for investigators. Eur Urol (2018) 74:796–804. doi: 10.1016/j.eururo.2018.08.038 30241973 PMC6261531

[40] Mandrekar JN. Receiver operating characteristic curve in diagnostic test assessment. J Thorac Oncol (2010) 5:1315–6. doi: 10.1097/JTO.0b013e3181ec173d 20736804

[41] Hänzelmann S Castelo R Guinney J. GSVA: Gene set variation analysis for microarray and RNA-seq data. BMC Bioinf (2013) 14:7. doi: 10.1186/1471-2105-14-7 PMC3618321 23323831

[42] Barbie DA Tamayo P Boehm JS Kim SY Moody SE Dunn IF. Systematic RNA interference reveals that oncogenic KRAS-driven cancers require TBK1. Nature (2009) 462:108–12. doi: 10.1038/nature08460 PMC2783335 19847166

[43] Charoentong P Finotello F Angelova M Mayer C Efremova M Rieder D. Pan-cancer immunogenomic analyses reveal genotype-immunophenotype relationships and predictors of response to checkpoint blockade. Cell Rep (2017) 18:248–62. doi: 10.1016/j.celrep.2016.12.019 28052254

[44] Newman AM Liu CL Green MR Gentles AJ Feng W Xu Y. Robust enumeration of cell subsets from tissue expression profiles. Nat Methods (2015) 12:453–7. doi: 10.1038/nmeth.3337 PMC4739640 25822800

[45] Mei Q Liu Z Zuo H Yang Z Qu J. Idiopathic pulmonary fibrosis: An update on pathogenesis. Front Pharmacol (2021) 12:797292. doi: 10.3389/fphar.2021.797292 35126134 PMC8807692

[46] Olson AL Gifford AH Inase N Fernández Pérez ER Suda T. The epidemiology of idiopathic pulmonary fibrosis and interstitial lung diseases at risk of a progressive-fibrosing phenotype. Eur Respir Rev (2018) 27(150):18077. doi: 10.1183/16000617.0077-2018 PMC9489016 30578336

[47] Morton GJ Cummings DE Baskin DG Barsh GS Schwartz MW. Central nervous system control of food intake and body weight. Nature (2006) 443:289–95. doi: 10.1038/nature05026 16988703

[48] Zhou R He M Fan J Li R Zuo Y Li B. The role of hypothalamic endoplasmic reticulum stress in schizophrenia and antipsychotic-induced weight gain: A narrative review. Front Neurosci (2022) 16:947295. doi: 10.3389/fnins.2022.947295 36188456 PMC9523121

[49] Hagimoto S Arima H Adachi K Ito Y Suga H Sugimura Y. Expression of neuropeptide Y and agouti-related protein mRNA stimulated by glucocorticoids is attenuated *via* NF-κB p65 under ER stress in mouse hypothalamic cultures. Neurosci Lett (2013) 553:165–9. doi: 10.1016/j.neulet.2013.08.040 23994062

[50] Frazzi R. BIRC3 and BIRC5: Multi-faceted inhibitors in cancer. Cell Biosci (2021) 11:8. doi: 10.1186/s13578-020-00521-0 33413657 PMC7792207

[51] Crawford N Stott KJ Sessler T McCann C McDaid W Lees A. Clinical positioning of the IAP antagonist Tolinapant (ASTX660) in colorectal cancer. Mol Cancer Ther (2021) 20:1627–39. doi: 10.1158/1535-7163.MCT-20-1050 PMC7611622 34389694

[52] Morón-Calvente V Romero-Pinedo S Toribio-Castelló S Plaza-Díaz J Abadía-Molina AC Rojas-Barros DI. Inhibitor of apoptosis proteins, NAIP, cIAP1 and cIAP2 expression during macrophage differentiation and M1/M2 polarization. PloS One (2018) 13:e0193643. doi: 10.1371/journal.pone.0193643 29518103 PMC5843221

[53] Du L Xu C Zeng Z Chen F Tang K Liang Y. Exploration of induced sputum BIRC3 levels and clinical implications in asthma. BMC Pulm Med (2022) 22:86. doi: 10.1186/s12890-022-01887-2 35287655 PMC8922789

[54] Li S Yang P Xu L Li M. Blocking of Birc3/TLR4/Myd88 signaling protects carbapenem-resistant Klebsiella pneumoniae in a mouse model of infection. Transpl Immunol (2021) 69:101464. doi: 10.1016/j.trim.2021.101464 34500040

[55] Zhang H Ma B Li N Zhang L Xu J Zhang S. SNHG1, a KLF4-upregulated gene, promotes glioma cell survival and tumorigenesis under endoplasmic reticulum stress by upregulating BIRC3 expression. J Cell Mol Med (2023) 27:1806–19. doi: 10.1111/jcmm.17779 PMC10315853 37243389

[56] Wu Y Lu S Huang X Liu Y Huang K Liu Z. Targeting cIAPs attenuates CCl4-induced liver fibrosis by increasing MMP9 expression derived from neutrophils. Life Sci (2022) 289:120235. doi: 10.1016/j.lfs.2021.120235 34914932

[57] Ajayi IO Sisson TH Higgins PDR Booth AJ Sagana RL Huang SK. X-linked inhibitor of apoptosis regulates lung fibroblast resistance to Fas-mediated apoptosis. Am J Respir Cell Mol Biol (2013) 49:86–95. doi: 10.1165/rcmb.2012-0224OC 23492187 PMC3727886

[58] Ashley SL Sisson TH Wheaton AK Kim KK Wilke CA Ajayi IO. Targeting inhibitor of apoptosis proteins protects from bleomycin-induced lung fibrosis. Am J Respir Cell Mol Biol (2016) 54:482–92. doi: 10.1165/rcmb.2015-0148OC PMC4821054 26378893

[59] Subramanian Vignesh K Deepe GS Jr. Metallothioneins: Emerging modulators in immunity and infection. Int J Mol Sci (2017) 18(10):2197. doi: 10.3390/ijms18102197 29065550 PMC5666878

[60] Liang T Zhang Q Sun W Xin Y Zhang Z Tan Y. Zinc treatment prevents type 1 diabetes-induced hepatic oxidative damage, endoplasmic reticulum stress, and cell death, and even prevents possible steatohepatitis in the OVE26 mouse model: Important role of metallothionein. Toxicol Lett (2015) 233:114–24. doi: 10.1016/j.toxlet.2015.01.010 25617602

[61] Yang L Hu N Jiang S Zou Y Yang J Xiong L. Heavy metal scavenger metallothionein attenuates ER stress-induced myocardial contractile anomalies: Role of autophagy. Toxicol Lett (2014) 225:333–41. doi: 10.1016/j.toxlet.2013.12.024 PMC4041391 24440343

[62] Dai H Wang L Li L Huang Z Ye L. Metallothionein 1: A new spotlight on inflammatory diseases. Front Immunol (2021) 12:739918. doi: 10.3389/fimmu.2021.739918 34804020 PMC8602684

[63] Sauler M McDonough JE Adams TS Kothapalli N Barnthaler T Werder RB. Characterization of the COPD alveolar niche using single-cell RNA sequencing. Nat Commun (2022) 13:494. doi: 10.1038/s41467-022-28062-9 35078977 PMC8789871

[64] Lin X Jagadapillai R Cai J Cai L Shao G Gozal E. Metallothionein induction attenuates the progression of lung injury in mice exposed to long-term intermittent hypoxia. Inflammation Res (2020) 69:15–26. doi: 10.1007/s00011-019-01287-z 31707449

[65] Zhong SC Luo X Chen XS Cai QY Liu J Chen XH. Expression and subcellular location of alpha-synuclein during mouse-embryonic development. Cell Mol Neurobiol (2010) 30:469–82. doi: 10.1007/s10571-009-9473-4 PMC11498774 19885730

[66] Abeliovich A Schmitz Y Fariñas I Choi-Lundberg D Ho WH Castillo PE. Mice lacking alpha-synuclein display functional deficits in the nigrostriatal dopamine system. Neuron (2000) 25:239–52. doi: 10.1016/s0896-6273(00)80886-7 10707987

[67] Stojkovska I Wani WY Zunke F Belur NR Pavlenko EA Mwenda N. Rescue of α-synuclein aggregation in Parkinson’s patient neurons by synergistic enhancement of ER proteostasis and protein trafficking. Neuron (2022) 110:436–451.e11. doi: 10.1016/j.neuron.2021.10.032 34793693 PMC8815333

[68] Xu B Wang F Wu SW Deng Y Liu W Feng S. Alpha-synuclein is involved in manganese-induced ER stress *via* PERK signal pathway in organotypic brain slice cultures. Mol Neurobiol (2014) 49:399–412. doi: 10.1007/s12035-013-8527-2 23934647

[69] Lyra P MaChado V Rota S Chaudhuri KR Botelho J Mendes JJ. Revisiting alpha-synuclein pathways to inflammation. Int J Mol Sci (2023) 24. doi: 10.3390/ijms24087137 PMC10138587 37108299

[70] Bozic M Caus M Rodrigues-Diez RR Pedraza N Ruiz-Ortega M Garí E. Protective role of renal proximal tubular alpha-synuclein in the pathogenesis of kidney fibrosis. Nat Commun (2020) 11:1943. doi: 10.1038/s41467-020-15732-9 32327648 PMC7181766

[71] Nygaard P. On the role of cytidine deaminase in cellular metabolism. Adv Exp Med Biol (1986) 195 Pt B:415–20. doi: 10.1007/978-1-4684-1248-2_65 3532704

[72] Tibaldi E Brocca A Sticca A Gola E Pizzi M Bordin L. Fam20C-mediated phosphorylation of osteopontin is critical for its secretion but dispensable for its action as a cytokine in the activation of hepatic stellate cells in liver fibrogenesis. FASEB J (2020) 34:1122–35. doi: 10.1096/fj.201900880R 31914633

[73] Saotome Y Winter CG Hirsh D. A widely expressed novel C2H2 zinc-finger protein with multiple consensus phosphorylation sites is conserved in mouse and man. Gene (1995) 152:233–8. doi: 10.1016/0378-1119(94)00717-7 7835706

[74] Allden SJ Ogger PP Ghai P McErlean P Hewitt R Toshner R. The transferrin receptor CD71 delineates functionally distinct airway macrophage subsets during idiopathic pulmonary fibrosis. Am J Respir Crit Care Med (2019) 200:209–19. doi: 10.1164/rccm.201809-1775OC PMC6635794 31051082

[75] Khalil N O’Connor RN Unruh HW Warren PW Flanders KC Kemp A. Increased production and immunohistochemical localization of transforming growth factor-beta in idiopathic pulmonary fibrosis. Am J Respir Cell Mol Biol (1991) 5:155–62. doi: 10.1165/ajrcmb/5.2.155 1892646

[76] Prasse A Pechkovsky DV Toews GB Jungraithmayr W Kollert F Goldmann T. A vicious circle of alveolar macrophages and fibroblasts perpetuates pulmonary fibrosis *via* CCL18. Am J Respir Crit Care Med (2006) 173:781–92. doi: 10.1164/rccm.200509-1518OC 16415274

[77] Reyfman PA Walter JM Joshi N Anekalla KR McQuattie-Pimentel AC Chiu S. Single-cell transcriptomic analysis of human lung provides insights into the pathobiology of pulmonary fibrosis. Am J Respir Crit Care Med (2019) 199:1517–36. doi: 10.1164/rccm.201712-2410OC PMC6580683 30554520

[78] García-Prieto E González-López A Cabrera S Astudillo A Gutiérrez-Fernández A Fanjul-Fernandez M. Resistance to bleomycin-induced lung fibrosis in MMP-8 deficient mice is mediated by interleukin-10. PloS One (2010) 5:e13242. doi: 10.1371/journal.pone.0013242 20949050 PMC2951918

[79] Hou J Shi J Chen L Lv Z Chen X Cao H. M2 macrophages promote myofibroblast differentiation of LR-MSCs and are associated with pulmonary fibrogenesis. Cell Commun Signal (2018) 16:89. doi: 10.1186/s12964-018-0300-8 30470231 PMC6260991

