# Supplementary figures and images for: Comprehensive analysis of an endoplasmic reticulum stress-related gene prediction model and immune infiltration in idiopathic pulmonary fibrosis
Source: Front Immunol. 2024 Jan 11;14:1305025. doi: 10.3389/fimmu.2023.1305025 (PMC10808546; doi:10.3389/fimmu.2023.1305025)

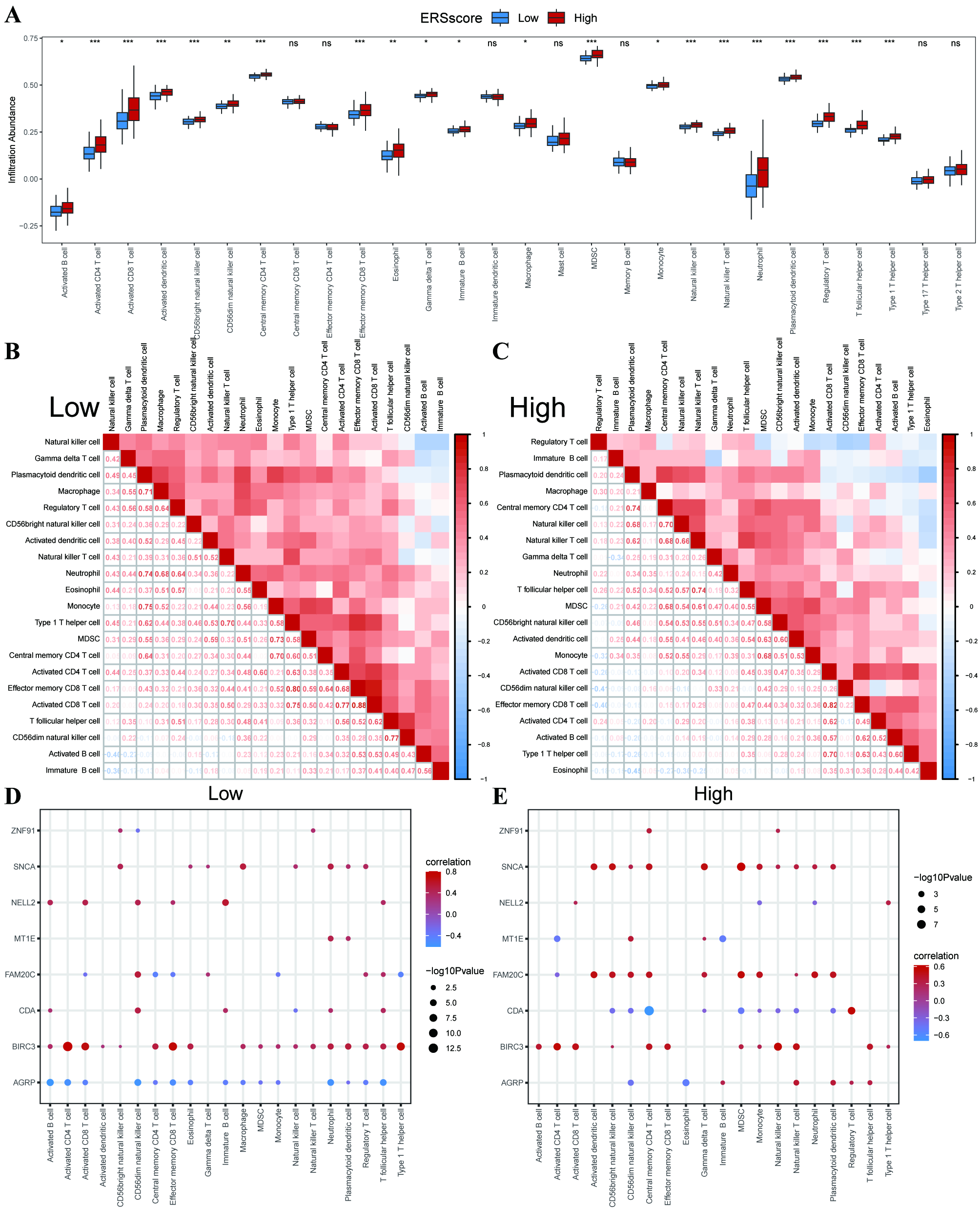

Supplement: Supplementary Figure 1 — Immune infiltration analysis evaluated between groups with high and low ERS scores by the ssGSEA algorithm in GSE70866 dataset. (A) The difference in expression of 28 immune cells between the high- and low-ERS score groups. (B, C) Correlation heatmap showed the correlation coefficient between different immune cells in the low-(B) and high-ERS(C) score groups. (D, E) The correlation analysis between common ERSRDEGs and specific immune cells in the low-(D) and high-ERS(E) score groups. “ns”, not significant (p-value >0.05). *p<0.05, **p<0.01, ***p<0.001. ssGSEA, single-sample gene-set enrichment analysis; ERS score, endoplasmic reticulum stress score; ERSRDEGs, endoplasmic reticulum stress-related differentially expressed genes. [file Image_1.tif]

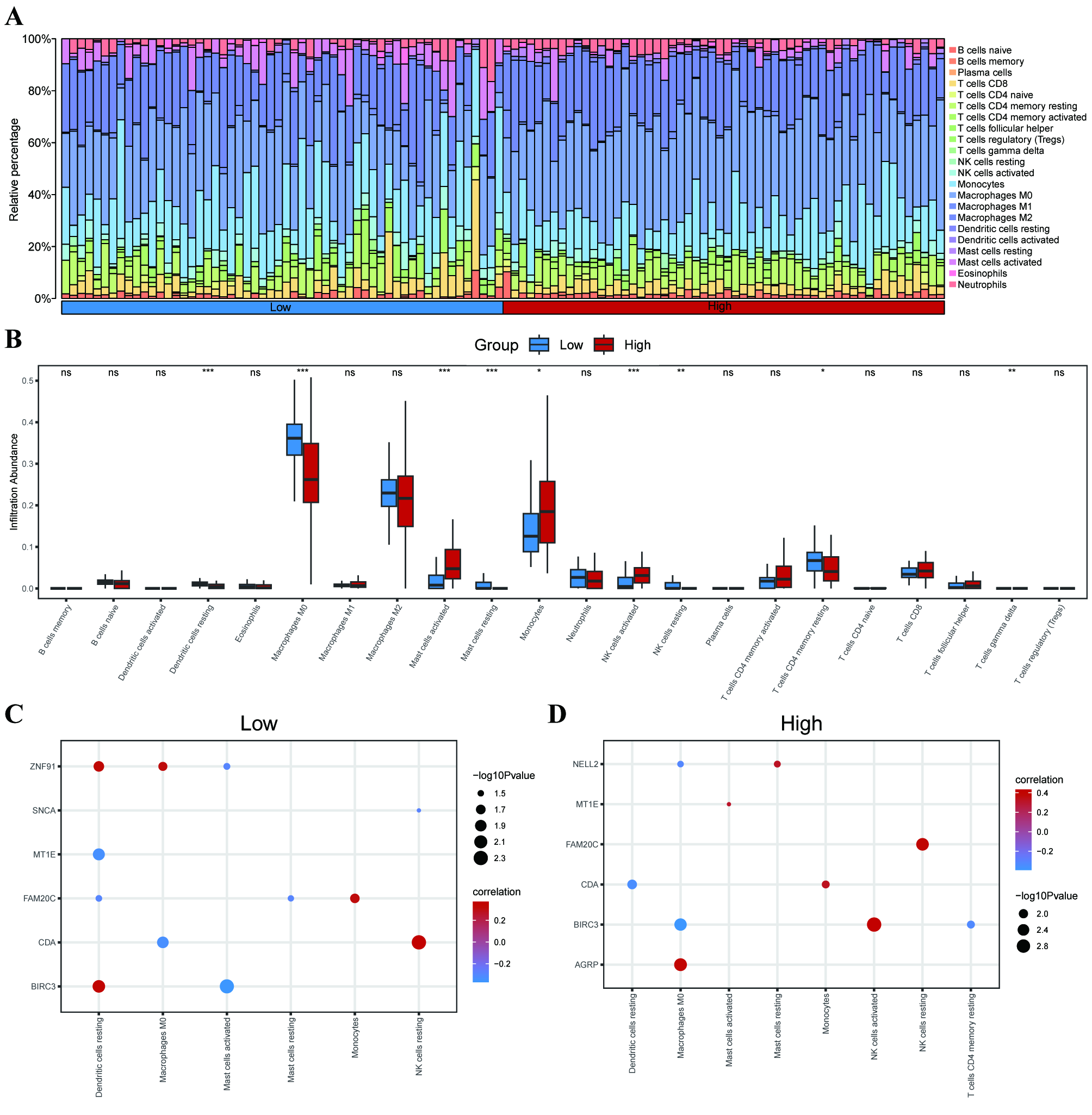

Supplement: Supplementary Figure 2 — Immune infiltration analysis evaluated between groups with high and low ERS scores by the CIBERSORT algorithm in GSE70866 dataset. (A) Histogram showing the distribution of 22 immune cell infiltration between the high- and low-ERS score groups. (B) Boxplot showing the differences in infiltrated immune cells between the high- and low-ERS score groups. (C, D) The correlation analysis between common ERSRDEGs and specific immune cells in the low-(C) and high-ERS(D) score groups. “ns”, not significant (p-value >0.05). *p<0.05, **p<0.01, ***p<0.001. ERS score, endoplasmic reticulum stress score; ERSRDEGs, endoplasmic reticulum stress-related differentially expressed genes. [file Image_2.tif]

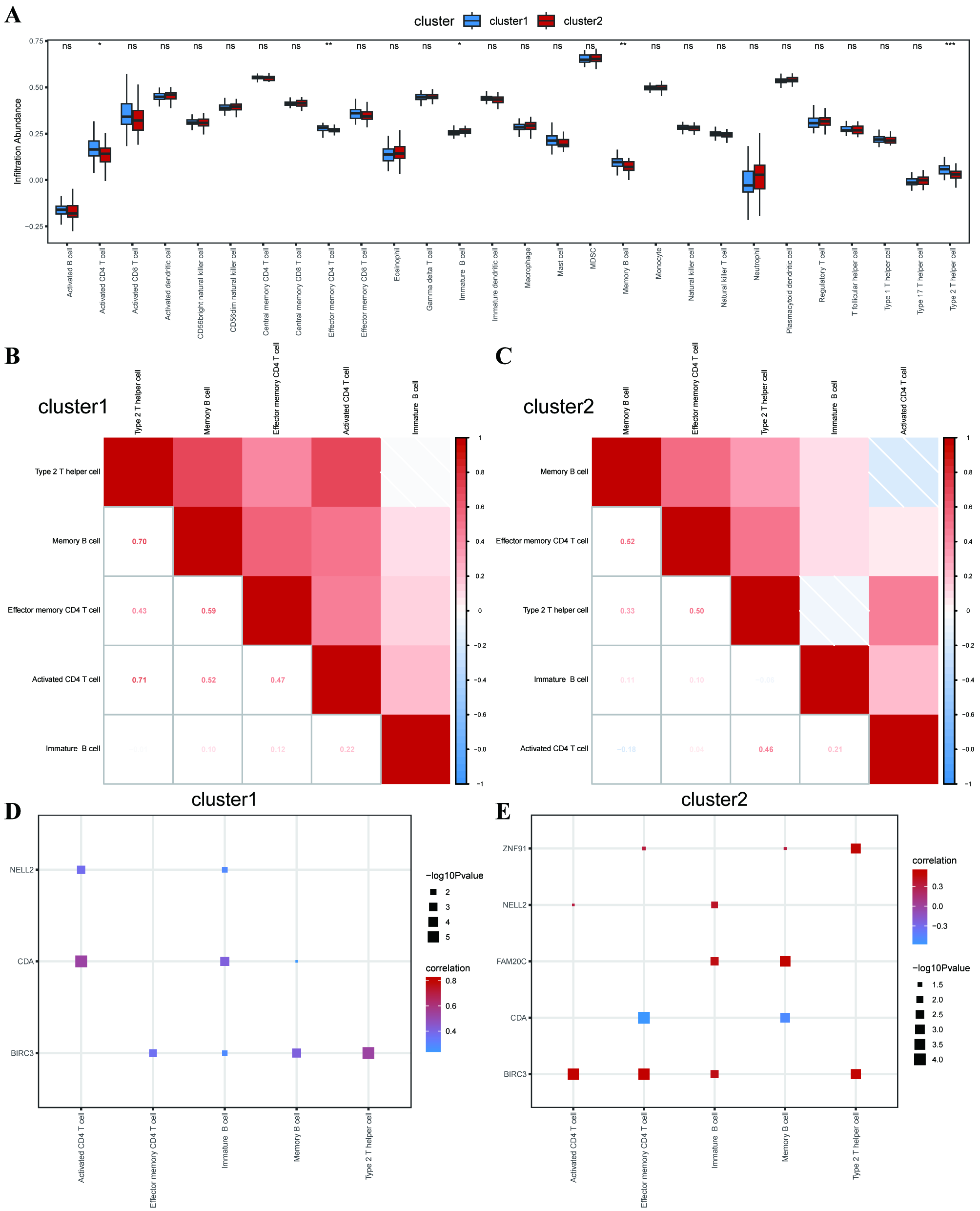

Supplement: Supplementary Figure 3 — Immune infiltration analysis evaluated between clusters 1 and 2 by the ssGSEA algorithm in GSE70866 dataset. (A) The difference in expression of 28 immune cells between clusters 1 and 2. (B, C) Correlation heatmap showed the correlation coefficient between different immune cells in clusters 1(B) and 2(C). (D, E) The correlation analysis between common ERSRDEGs and specific immune cells in clusters 1 (D) and 2 (E). “ns”, not significant (p-value >0.05). *p<0.05, **p<0.01, ***p<0.001. ssGSEA, single-sample gene-set enrichment analysis; ERSRDEGs, endoplasmic reticulum stress-related differentially expressed genes. [file Image_3.tif]

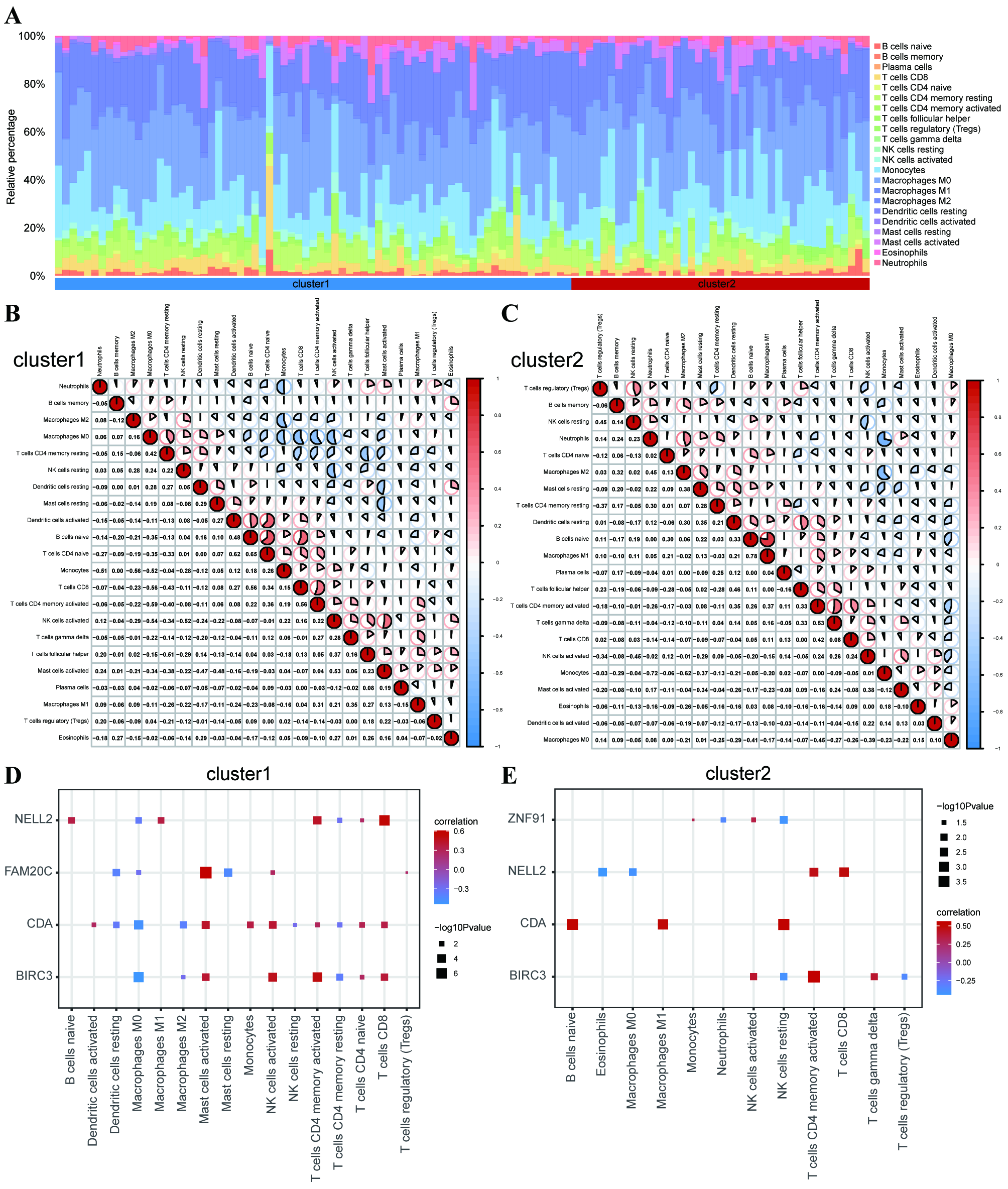

Supplement: Supplementary Figure 4 — Immune infiltration analysis evaluated between clusters 1 and 2 by the CIBERSORT algorithm in GSE70866 dataset. (A) Histogram showing the distribution of 22 immune cell infiltration between clusters 1 and 2. (B, C) Correlation heatmap showed the correlation coefficient between different immune cells in clusters 1(B) and 2(C). (D, E) The correlation analysis between common ERSRDEGs and specific immune cells in clusters 1 (D) and 2 (E). *p<0.05, **p<0.01, ***p<0.001. ERSRDEGs, endoplasmic reticulum stress-related differentially expressed genes. [file Image_4.tif]
