# Supplementary material for: Comprehensive analysis of an endoplasmic reticulum stress-related gene prediction model and immune infiltration in idiopathic pulmonary fibrosis
Source: Front Immunol. 2024 Jan 11;14:1305025. doi: 10.3389/fimmu.2023.1305025 (PMC10808546; doi:10.3389/fimmu.2023.1305025)
Supplement: Supplementary file 6 [file Table_2.docx]

### Table S2. List of external validation data sets for idiopathic pulmonary fibrosis.

|  | **GSE24206** | **GSE93606** |
| --- | --- | --- |
| Platform | GPL570 | GPL11532 |
| Species | Homo sapiens | Homo sapiens |
| Tissue | lung tissue | bronchoalveolar lavage cell and peripheral blood mononuclear cell |
| Samples in IPF group | 17 | 154 |
| Samples in Control group | 6 | 20 |
| Reference | Bayesian probit regression model for the diagnosis of pulmonary fibrosis: proof-of-principle | Host-Microbial Interactions in Idiopathic Pulmonary Fibrosis |

IPF，Idiopathic Pulmonary Fibrosis；GEO，Gene Expression Omnibus。
