# Supplementary material for: Comprehensive analysis of an endoplasmic reticulum stress-related gene prediction model and immune infiltration in idiopathic pulmonary fibrosis
Source: Front Immunol. 2024 Jan 11;14:1305025. doi: 10.3389/fimmu.2023.1305025 (PMC10808546; doi:10.3389/fimmu.2023.1305025)
Supplement: Supplementary file 7 [file Table_3.docx]

Table S3. RT-qPCR primers used for gene expression analysis

| Gene name | Human primers | Mouse primers |
| --- | --- | --- |
| AGRP | F: TGCGTAAGGCTGCATGAGTCC  R: GCATTGAAGAAGCGGCAGTAGC | F: GCAGACCGAGCAGAAGAAGTTC  R: GGCATTGAAGAAGCGGCAGTAG |
| BIRC3 | F: TGTGATGGTGGACTCAGGTGTTG  R: CTGGCTTGAACTTGACGGATGAAC | F: CTTTGCGTGCGATGGGAAACTG  R: AATGCCTCTGGTGCTCTGACATAG |
| CDA | F:CCAGGAGGGGAGAATCTTCAAAGG  R: ATAGCGGTCCGTTCAGCACAG | F: AGGAACGCCCCTCCTGTG  R: GACTTCTTGGCCTCACGAGAG |
| FAM20C | F: GACTCCTATCCCAACTGGCTCAAG  R: CTCGATGGCCGGGTTGTGTC | F: TTCTCGCTCCTCTTCATCAGTGC  R: GGCTCAGTTTGTGTTCCTCTTTGG |
| MT1E | F: ATGGACCCCAACTGCTCTTGC  R: GCACTCTTTGCACTTGCAGGAG | F: CCCAACTGCTCCTGCTCCAC  R: ACAGCCCTGGGCACATTTGG |
| NELL2 | F: CCCTGCCTTGCCCAGATGTG  R: GTCTTGGTGATGTCATTGCGGATG | F: CACTACCGCTCTGGCACTCAC  R: GAGAGGCACTGAAGGCTAAGGAG |
| SNCA | F: TTCAAAGGCCAAGGAGGGAGTTG  R: TTCCTGCTGCTTCTGCCACAC | F: CAAGCAGGGTGTGGCAGAGG  R: TGTCACTCCATGAACCACTCCTTC |
| ZNF91 | F:GAGGATACACACTGGAGAGAAGCC  R: TGCTTAGTAAGGGTTGAGGAACGG | F: AGAGGGAGAAGGCGAAAAGATGAC  R: ATAGCGAGGGTGAGCAAGAACAG |
| GAPDH | F: CAGGAGGCATTGCTGATGAT  R: GAAGGCTGGGGCTCATTT | F: GGTTGTCTCCTGCGACTTCA  R: TGGTCCAGGGTTTCTTACTCC |
